# Effects of genetic variants in the TSPO gene on protein structure and stability

**DOI:** 10.1371/journal.pone.0195627

**Published:** 2018-04-11

**Authors:** Vladimir M. Milenkovic, Stefanie Bader, Daniel Sudria-Lopez, Ramona Siebert, Caroline Brandl, Caroline Nothdurfter, Bernhard H. F. Weber, Rainer Rupprecht, Christian H. Wetzel

**Affiliations:** 1 Department of Psychiatry and Psychotherapy, Molecular Neurosciences, University of Regensburg, Regensburg, Germany; 2 Institute of Human Genetics, University of Regensburg, Regensburg, Germany; 3 Department of Ophthalmology, University Hospital Regensburg, Regensburg, Germany; University of Michigan, UNITED STATES

## Abstract

The 18 kDa translocator protein (TSPO) is an evolutionary conserved cholesterol binding protein localized in the outer mitochondrial membrane. Expression of TSPO is upregulated in activated microglia in various neuroinflammatory, neurodegenerative, and neoplastic disorders. Therefore, TSPO radioligands are used as biomarkers in positron emission tomography (PET) studies. In particular, a common A147T polymorphism in the TSPO gene affects binding of several high affinity TSPO radioligands. Given the relevance of TSPO as a diagnostic biomarker in disease processes, we systematically searched for mutations in the human TSPO gene by a wide array of evolution and structure based bioinformatics tools and identified potentially deleterious missense mutations. The two most frequently observed missense mutations A147T and R162H were further analysed in structural models of human wildtype and mutant TSPO proteins. The effects of missense mutations were studied on the atomic level using molecular dynamics simulations. To analyse putative effects of A147T and R162H variants on protein stability we established primary dermal fibroblast cultures from wt and homozygous A147T and R162H donors. Stability of endogenous TSPO protein, which is abundantly expressed in fibroblasts, was studied using cycloheximide protein degradation assay. Our data show that the A147T mutation significantly alters the flexibility and stability of the mutant protein. Furthermore both A147T and R162H mutations decreased the half-life of the mutant proteins by about 25 percent, which could in part explain its effect on reduced pregnenolone production and susceptibility to neuropsychiatric disorders. The present study is the first comprehensive bioinformatic analysis of genetic variants in the TSPO gene, thereby extending the knowledge about the clinical relevance of TSPO nsSNPs.

## Introduction

The translocator protein 18 kDa (TSPO) is an 18 kDa evolutionary conserved cholesterol binding protein localized in the outer mitochondrial membrane (OMM) [1]. TSPO is widely expressed in many tissues with highest expression levels in steroid-synthesizing cells of endocrine organs [2, 3]. TSPO has been implicated in various cellular processes including steroid hormone biosynthesis, oxidative stress, cell proliferation and apoptosis [4–6]; although recent studies applying conditional and global knock out mice challenged to what extent TSPO is essential for survival and steroid biosynthesis [7, 8].

TSPO expression which is low in healthy brain, is upregulated in neuroinflammation and neurodegenerative disorders, such as Alzheimer disease, Parkinson disease, multiple sclerosis and stroke [4, 9], but also during major depressive episodes [10]. Consequently, radioligands that bind TSPO have been used in positron emission tomography (PET) studies [11] as a biomarker of neuroinflammation. In these studies, it has been shown that binding affinity of TSPO PET radiotracers such as ^11^C-PBR28 is greatly affected by the non-synonymous rs6791 polymorphism [12], which is present in about 30% of the Caucasian population. The polymorphism was associated with predisposition to neuropsychiatric disorders such as panic disorder [13], adult separation anxiety [14], bipolar disease [15], and fibromyalgia [16], as well as a reduced pregnenolone production by peripheral blood cells [17].

Given the importance of TSPO as a biomarker for neuroinflammation and the potential therapeutic implication of new TSPO ligands, we sought to systematically investigate genetic variants in TSPO by several computational methods. Comparing the results of eight evolution and structure based prediction tools used, we identified the most deleterious variants in the TSPO gene. Interestingly, half of the identified deleterious missense mutations cluster in the evolutionary highly conserved transmembrane domains 2 and 5, which are facing each other. To study the impact of the two most frequently observed genetic variants, namely A147T and R162H, at the structural level, models of the mutant proteins were built by homology modelling based on the *Bacillus cereus* TSPO crystal structure. Further, the native and mutant proteins were analysed applying molecular dynamics experiments to determine whether the two polymorphic sites could affect TSPO protein structure and stability. In addition, stability of wt and mutant proteins was analysed in vitro in fibroblast cell lines carrying the respective genotypes. This study is a first extensive in silico analysis of the TSPO gene serving as a resource for future structure-function analyses of the TSPO protein.

## Results

### SNP dataset collection

**Fig 1:**
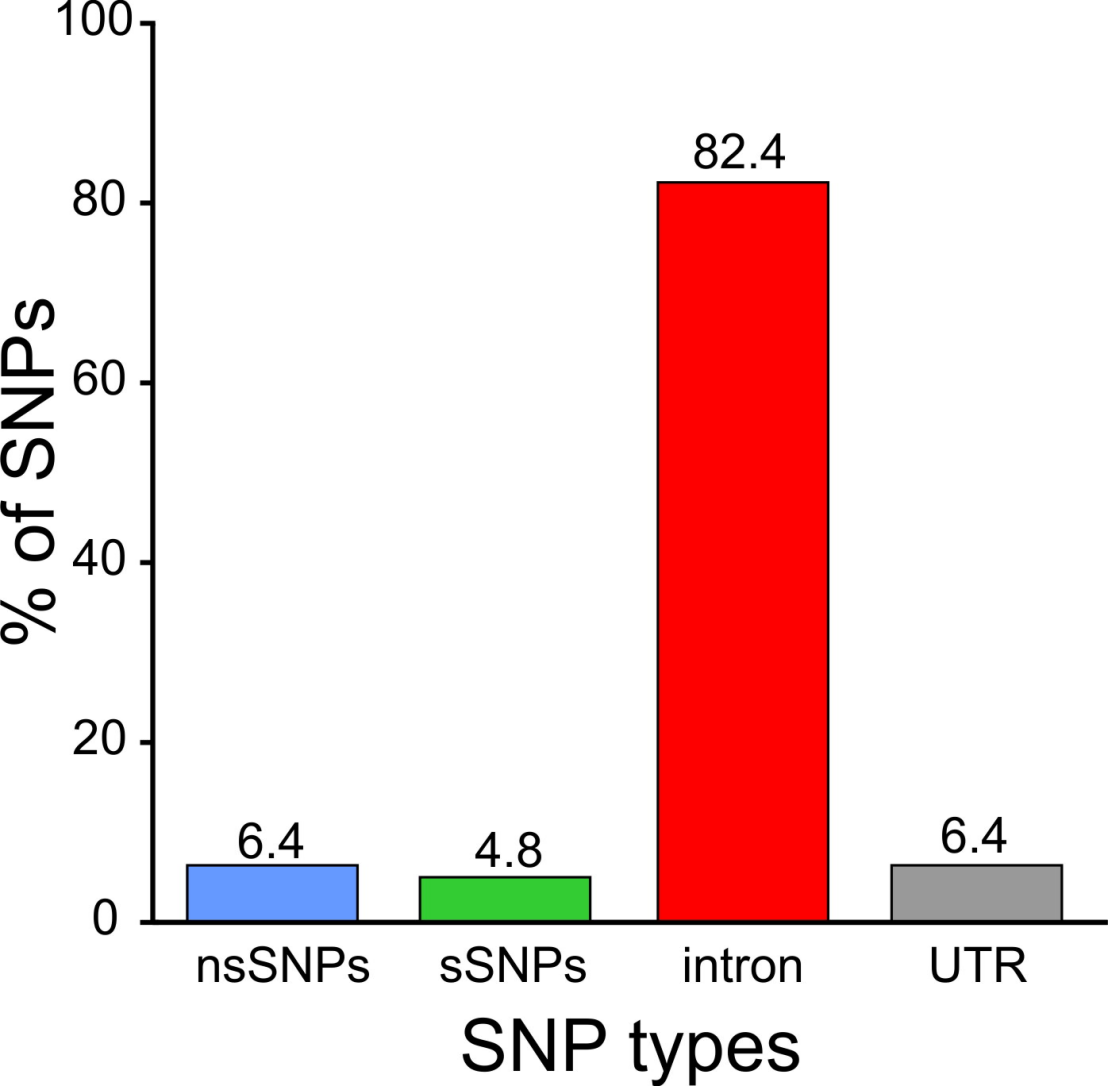
Distribution of coding nsSNPs, coding sSNPs, intronic SNPs, and UTR SNPs in the human TSPO gene.

**Fig 2:**
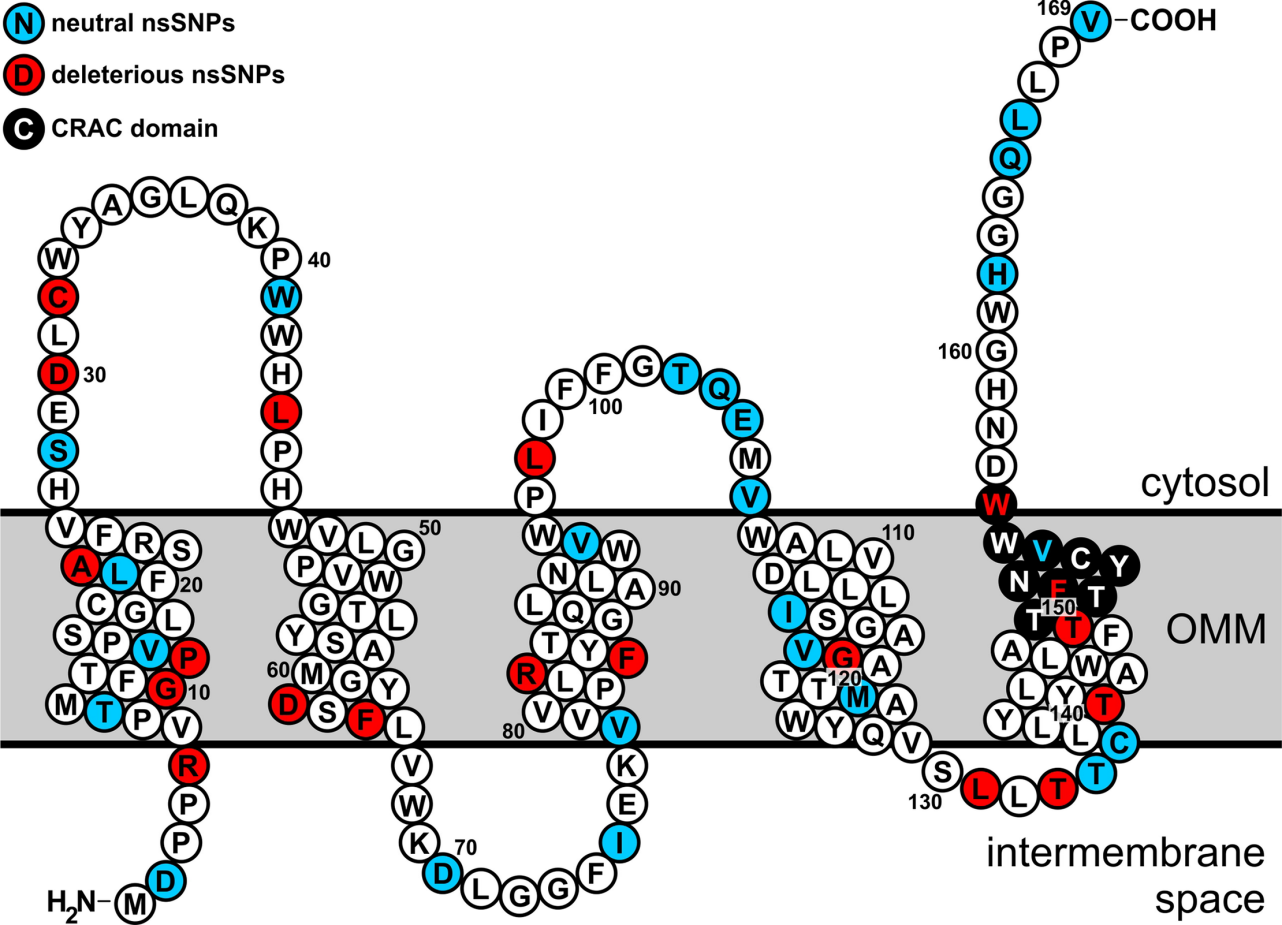
Identification of deleterious nsSNPs in human TSPO gene. Out of 52 nsSNPs analysed using a wide array of sequence and structure based computational methods, 21 were predicted to be deleterious. Non-deleterious (blue), and deleterious (red), nsSNPs were plotted onto topology model of human TSPO which was generated with PROTTER [18]. Cholesterol recognition/interaction amino acid consensus sequence (CRAC) domain is highlighted in black.

The human TSPO gene reveals a total of 818 genetic variants in a data set from dbSNP and 60706 unrelated individuals from Exome Aggregation Consortium (ExAC) (Fig 1 and S1 Table). Of these, the majority is in the non-coding intervening regions of the gene (674 variants; 82.4%), and 52 (6.4%) genetic variants in the 5’- and 3’-untranslated regions of the mRNA transcript. Genetic variants in the coding region include 52 (6.4%) non-synonymous SNPs (nsSNPs) and 40 (4.8%) synonymous SNPs (sSNPs), which are evenly distributed across the protein coding sequence. The nsSNPs were mapped to 43 residues (Fig 2), with 9 amino acid residues harbouring two mutations each (G63S/D, A120P/G, R135C/H, P139T/S, A147T/M, R156W/Q, R165W/Q, R166W/L, and E169Q/V). Since we sought to assess the effect of the mutations on protein stability and structure, all synonymous and non-synonymous SNPs were selected for subsequent analysis.

### Identification of deleterious SNPs in TSPO gene

Computational methods such as Provean, SIFT, Polyphen2, PhD-SNP, SNAP2, SNPs&GO, FATHMM and I-Mutant 3 were used to investigate the effect of mutations in the TSPO gene on protein structure and stability (Table 1, S1 Table). Out of 52 nsSNPs analysed, 21 were predicted to be deleterious (Fig 2, Table 1) by consensus of all eight algorithms used. The evolutionary conservation prediction tool ConSurf revealed that 11 out of 21 of the predicted deleterious mutations occur in highly conserved regions of the gene (S1 Fig, S2 Table). Interestingly, 9 out of these 11 mutations are located in two adjacent, evolutionary conserved amino acid stretches in transmembrane domain 2 (TMD) (aa 44–65) and in TMD5 (aa 133–150) (S1 Fig), suggesting their functional importance. Because all of the nsSNPs, except A147T, and R162H have a very low global minor allele frequencies (MAF) in human populations (MAF<0.003), we have selected these two SNPs (A147T and R162H) for further analysis. A147T SNP (MAF 0.30 in European and 0.05 in Asian populations) affects binding of the second generation of the TSPO ligands, and has been shown to be associated with anxiety disorder [14] and lowered production of pregnenolone [17]. Functional consequences of the R162H SNP with a global MAF of 0.30 were not reported so far, so we also included this SNP in further analysis.

**Table 1:** List of nsSNPs in TSPO gene and their predicted Provean, SIFT, PolyPhen2, PhD-SNP, SNAP2, SNPs&GP, FATHMM, and iMutant3 scores. (N = non-deleterious, D = deleterious).

	SNP ID	Position	Provean	SIFT	PolyPhen2	PhD-SNP	SNAP2	SNPs&GO	FATHMM	iMutant3	Consensus 1–4 = N, >5 = D
1	rs764229144	A2D	Neutral	Damaging	possibly damaging	Neutral	Neutral	Neutral	Tolerated	Neutral	N
2	rs753849621	W5R	Deleterious	Damaging	probably damaging	Neutral	Effect	Neutral	Tolerated	Large decrease of stability	**D**
3	rs765728030	A8T	Neutral	Tolerated	probably damaging	Neutral	Neutral	Neutral	Tolerated	Large decrease of stability	N
4	rs567132675	G10S	Deleterious	Damaging	probably damaging	Neutral	Effect	Neutral	Tolerated	Large decrease of stability	**D**
5	rs758845609	L13P	Deleterious	Damaging	probably damaging	Disease	Effect	Neutral	Damaging	Large decrease in stability	**D**
6	rs187866832	A14V	Neutral	Tolerated	benign	Neutral	Neutral	Neutral	Tolerated	Neutral	N
7	rs777431441	V21L	Neutral	Tolerated	benign	Neutral	Neutral	Neutral	Tolerated	Large decrease in stability	N
8	rs748778969	G22A	Deleterious	Damaging	benign	Disease	Effect	Neutral	Tolerated	Large decrease in stability	**D**
9	rs778946143	G28S	Neutral	Tolerated	probably damaging	Neutral	Neutral	Neutral	Tolerated	Large decrease in stability	N
10	rs775263818	G30D	Neutral	Damaging	probably damaging	Disease	Effect	Neutral	Tolerated	Large decrease in stability	**D**
11	rs566580110	R32C	Neutral	Tolerated	probably damaging	Disease	Effect	Neutral	Damaging	Large decrease in stability	**D**
12	rs750994845	S41W	Deleterious	Tolerated	benign	Disease	Neutral	Disease	Damaging	Neutral	N
13	rs550303992	P44L	Deleterious	Damaging	probably damaging	Disease	Effect	Disease	Damaging	Neutral	**D**
14	rs566547284	G63S	Neutral	Tolerated	probably damaging	Disease	Effect	Neutral	Tolerated	Large decrease in stability	**D**
15	rs139234976	G63D	Deleterious	Damaging	probably damaging	Neutral	Effect	Disease	Tolerated	Large decrease in stability	**D**
16	rs756858058	Y65F	Deleterious	Damaging	probably damaging	Disease	Effect	Neutral	Tolerated	Large decrease in stability	**D**
17	rs199899658	E70D	Neutral	Tolerated	possibly damaging	Neutral	Neutral	Neutral	Tolerated	Neutral	N
18	rs749849080	T75I	Deleterious	Damaging	possibly damaging	Neutral	Neutral	Neutral	Tolerated	Neutral	N
19	rs372235648	A78V	Deleterious	Damaging	probably damaging	Neutral	Effect	Neutral	Tolerated	Neutral	N
20	rs746919529	G83R	Deleterious	Damaging	probably damaging	Disease	Effect	Disease	Tolerated	Neutral	**D**
21	rs754824182	L84F	Deleterious	Tolerated	probably damaging	Disease	Effect	Neutral	Damaging	Large decrease in stability	**D**
22	rs142445069	A94V	Deleterious	Damaging	possibly damaging	Disease	Effect	Neutral	Tolerated	Neutral	N
23	rs775043588	P97L	Deleterious	Damaging	probably damaging	Disease	Effect	Neutral	Tolerated	Neutral	**D**
24	rs760110771	A102T	Neutral	Tolerated	benign	Neutral	Neutral	Neutral	Tolerated	Neutral	N
25	rs775654599	R103Q	Neutral	Damaging	possibly damaging	Neutral	Effect	Neutral	Tolerated	Large decrease of stability	N
26	rs761543515	Q104E	Neutral	Tolerated	benign	Neutral	Effect	Neutral	Tolerated	Neutral	N
27	rs143915407	G106V	Deleterious	Tolerated	probably damaging	Disease	Effect	Neutral	Tolerated	Neutral	N
28	rs752645452	V115I	Neutral	Damaging	benign	Neutral	Neutral	Neutral	Tolerated	Large decrease of stability	N
29	rs148614502	A119V	Neutral	Tolerated	probably damaging	Neutral	Neutral	Neutral	Tolerated	Neutral	N
30	rs200880548	A120P	Deleterious	Damaging	possibly damaging	Neutral	Neutral	Neutral	Damaging	Neutral	N
31	rs779150979	A120G	Deleterious	Tolerated	benign	Disease	Effect	Disease	Tolerated	Large decrease of stability	**D**
32	rs780467525	V124M	Neutral	Tolerated	possibly damaging	Neutral	Neutral	Neutral	Tolerated	Large decrease of stability	N
33	rs373738253	P131L	Deleterious	Tolerated	probably damaging	Disease	Effect	Disease	Tolerated	Large decrease of stability	**D**
34	rs773881998	A133T	Deleterious	Damaging	probably damaging	Disease	Effect	Disease	Damaging	Large decrease of stability	**D**
35	rs767027529	A134T	Neutral	Tolerated	possibly damaging	Disease	Neutral	Neutral	Tolerated	Large decrease of stability	N
36	rs775344095	R135C	Neutral	Tolerated	benign	Disease	Neutral	Neutral	Tolerated	Large decrease of stability	N
37	rs760654235	R135H	Neutral	Tolerated	probably damaging	Neutral	Neutral	Neutral	Tolerated	Large decrease of stability	N
38	rs757578697	P139T	Deleterious	Damaging	probably damaging	Disease	Effect	Disease	Damaging	Large decrease of stability	**D**
39	n/a	P139S	Deleterious	Damaging	probably damaging	Disease	Effect	Disease	Damaging	Large decrease of stability	**D**
40	rs6971	A147T	Deleterious	Damaging	benign	Disease	Effect	Neutral	Tolerated	Large decrease of stability	**D**
41	rs141002863	A147M	Deleterious	Damaging	benign	Disease	Effect	Neutral	Tolerated	Neutral	N
42	rs774036527	L150F	Deleterious	Damaging	probably damaging	Disease	Effect	Disease	Damaging	Large decrease of stability	**D**
43	rs771545866	V154I	Neutral	Tolerated	benign	Neutral	Neutral	Neutral	Tolerated	Large decrease of stability	N
44	rs775001391	R156W	Deleterious	Damaging	probably damaging	Disease	Effect	Neutral	Tolerated	Neutral	**D**
45	rs375211541	R156Q	Neutral	Tolerated	benign	Disease	Effect	Neutral	Tolerated	Large decrease of stability	N
46	rs6972	R162H	Neutral	Tolerated	benign	Neutral	Effect	Neutral	Tolerated	Large decrease of stability	N
47	rs776603192	R165W	Neutral	Damaging	probably damaging	Neutral	Neutral	Neutral	Damaging	Neutral	N
48	rs761740678	R165Q	Neutral	Tolerated	probably damaging	Neutral	Effect	Neutral	Tolerated	Large decrease of stability	N
49	rs765102690	R166W	Neutral	Damaging	probably damaging	Neutral	Effect	Neutral	Damaging	Neutral	N
50	rs8192467	R166L	Neutral	Damaging	benign	Neutral	Effect	Neutral	Tolerated	Neutral	N
51	rs9333342	E169Q	Neutral	Damaging	possibly damaging	Neutral	Effect	Neutral	Tolerated	Neutral	N
52	rs568411305	E169V	Neutral	Damaging	possibly damaging	Neutral	Effect	Neutral	Tolerated	Neutral	N

### Analysis of sSNPs

Since it has been shown that the sSNPs could also affect protein translation efficiency and protein folding [19], we analysed codon usage of all 40 TSPO sSNPs using graphic codon usage analyser accessible under http://gcua.schoedl.de/ [20]. Unexpectedly, 3 of the 40 sSNPs showed significantly altered codon usage frequencies (S3 Table), expressed as relative adaptiveness which consider the number of codons that code for the respective amino acid. Relative adaptiveness values for the SNPs at positions 113, 114, and 141 with CTG changed to CTA (both encode Leu) were reduced from 100 to 18. The sSNPs at positions 113 and 114 are of special interest, as they reside in a rare codon usage cluster located between amino acids 100 and 121 (S2 Fig). Consequently, changes in the cluster of infrequently used codons could affect the timing of co-translational folding [21].

### Homology modelling of human wt and mutant TSPO molecules

Regardless of remarkable progress in experimentally solving protein structures, the structure of the human TSPO has not yet been determined. Only recently, the TSPO structures from two bacterial species has been resolved at atomic resolution [22, 23], together with the structure of mouse TSPO, which has been determined by NMR [24]. These data can now be used for homology modelling of native and mutant human TSPO proteins. The crystal structure of *Bacillus cereus* TSPO with a protein sequence identity of 26.4% served as a template for the homology modelling. The regions which could not be aligned to *Bacillus cereus* TSPO were additionally modelled including the loops containing the sequences MAPPWV, FTEK, and HGWRGGRRLP. Subsequently, the data were subjected to a combined steepest descent and simulated annealing minimization. The initial wt and the mutant A147T and R162H structures were further refined and subjected to an energy minimization algorithm.

**Fig 3:**
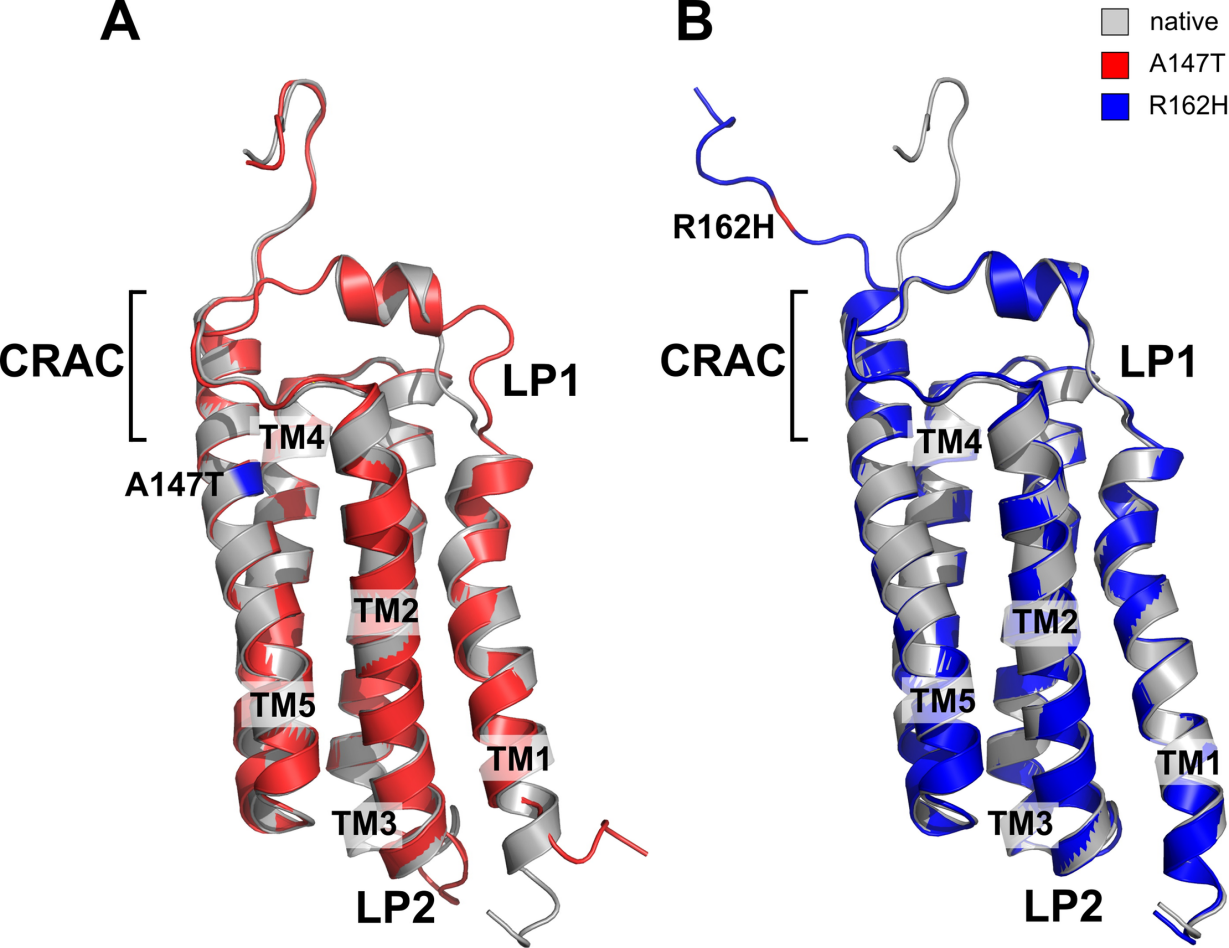
Spatial superposition of the native and mutant TSPO structures. Native human TSPO structure depicted in grey, was aligned with **(A)** A147T (red), and **(B)** R162H (blue) TSPO mutant structures, respectively. Mutation at position A147T destabilized the whole protein, especially the loop LP1, which is involved in ligand binding pocket and loop LP2. Mutation R162H, on the other hand, affected the conformation of the C-terminus only.

The refined models of native and mutant TSPO structures were superimposed to infer possible structural consequences of A147T and R162H mutations (Fig 3). The R162H substitution resulted in local changes at the C terminus of TSPO molecule (Fig 3B), while the A147T mutation (Fig 3A) caused considerable structural changes in TMD1, and the loop region 1 (LP1).

### Molecular dynamics simulation of native and mutant TSPO proteins

**Fig 4:**
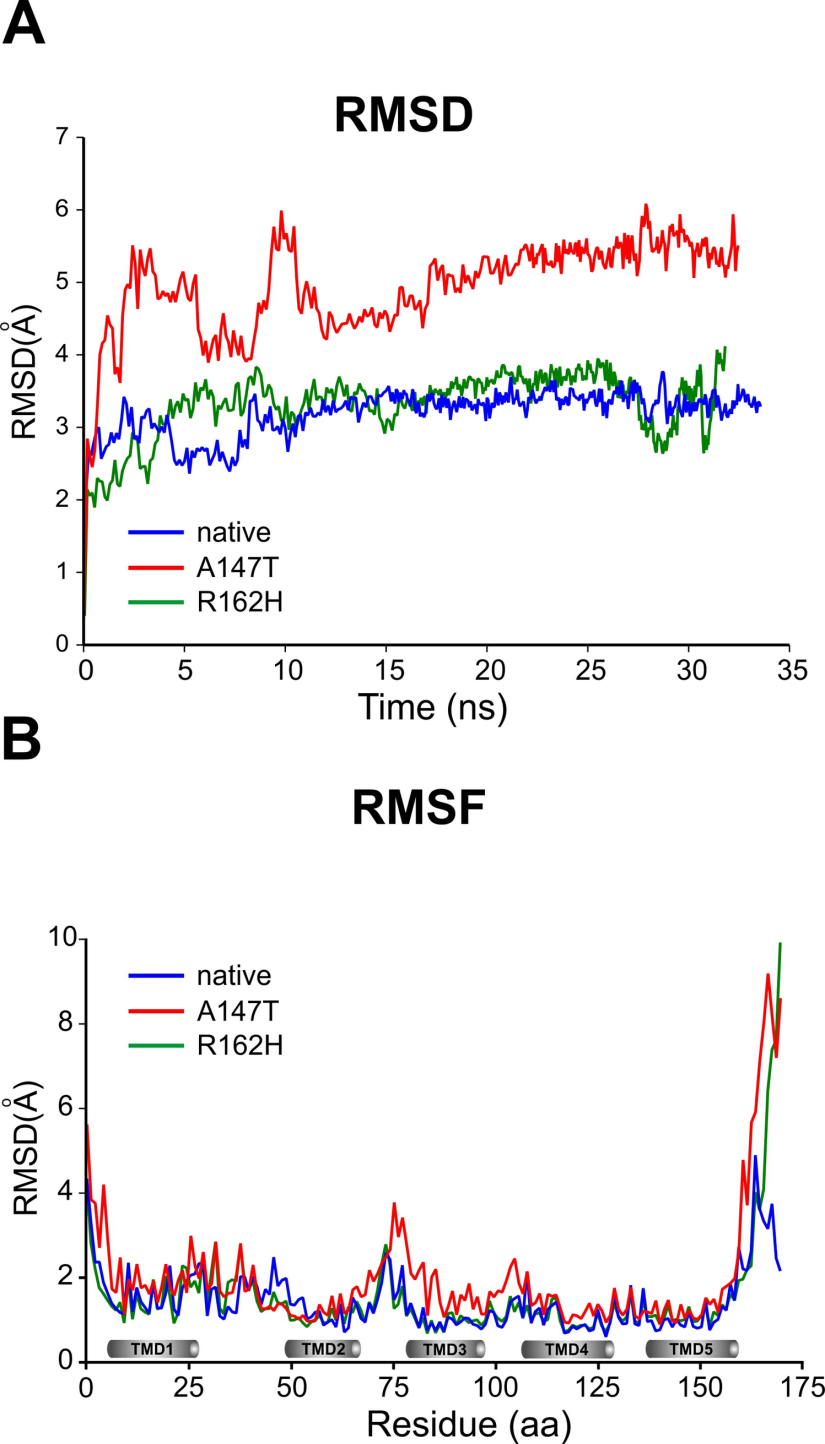
Backbone RMSD and per-residue RMSF values of native and mutant structures of the TSPO protein. **(A)** The RMSD values of native and mutant structures are shown as a function of time. **(B)** Central alpha carbon RMSF per residue of native, and mutant systems are shown. Transmembrane domains are depicted with grey cylinders. Blue, red and green lines indicate native, A147T and R162H mutations, respectively.

To further understand the consequences of the missense mutations on TSPO structure and stability, we conducted molecular dynamics simulation experiments using refined native and mutant protein structures. We analysed the root mean square deviation (RMSD), root mean square fluctuation (RMSF), and radius of gyration (Rg) between native and mutant molecules. Two independent simulations were carried out for each wt and mutant structures, respectively, and RMSD, RMSF, and Rg values were calculated from trajectory files. The overall protein stability changes were assessed with the help of backbone RMSD values, which were calculated for both native and mutant structures of the TSPO protein. Native and R162H structures reached equilibrium states after 12 ns of simulation (3.27±0.29Å, and 3.41±0.43Å respectively) (Fig 4A), whereas RMSD of the A147T mutant was significantly higher (5.14±0.63Å) during the complete simulation period (Table 2). This increased structural deviation reflects the impact of the amino acid substitution on protein stability. The A147T mutant results in a conformational change in protein structure which makes the backbone more flexible, and in turn could affect ligand binding.

**Table 2:** Average structural properties calculated for full length wt, A147T, and R162H TSPO models and corresponding standard deviations (in parentheses).

	TSPO wt	A147T	R162H
Backbone RMSD (Å)	3.27 (0.29)	5.14* (0.63)	3.41 (0.43)
Cα-RMSD (Å)	3.24 (0.30)	5.03* (0.61)	3.36 (0.43)
Cα-RMSF (Å)	1.43 (0.70)	2.00* (1.45)	1.51 (1.17)
Rg-protein (Å)	18.18	18.08	18.58

* Significant at p<0.05.

To evaluate the overall structural flexibility of native and mutant TSPO structures, RMSF values were calculated from the simulation trajectory files, and were plotted per amino acid residue (Fig 4B). The RMSFs of the A147T mutant structure (2.0±1.45Å) were notably higher (Table 2) than that of wild-type, or R162H variant (1.43±0.7Å, and 1.51±1.17Å, respectively) especially in the loop regions, thus suggesting greater structural flexibility of the mutant structure. Rg, which is the measure of compactness of protein structure and is defined as mass weighted root mean square distance of atoms from their centre of mass, did not differ between native and mutant structures (Table 2), suggestive of their similar overall protein dimensions.

In conclusion, our MDS results suggest that both A147T and R162H mutations cause a significant structural change, resulting in higher overall flexibility when compared to the native protein, which could affect binding affinity of TSPO ligands.

### Analysis of protein stability

**Fig 5:**
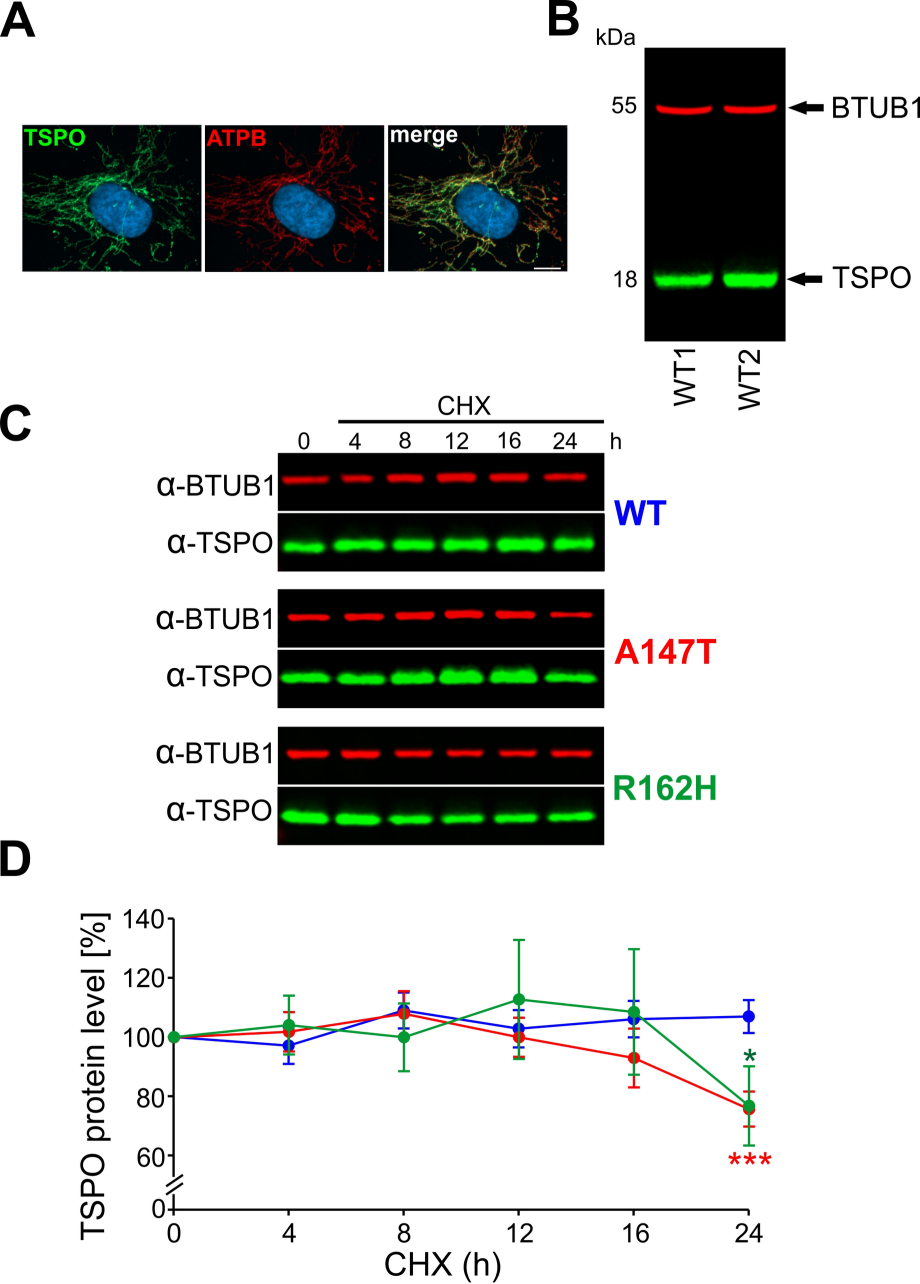
Analysis of protein stability by the cycloheximide protein degradation assay of wt and mutant TSPO proteins. **(A)** TSPO is localized to mitochondria in primary dermal fibroblasts. **(B)** Lysates of human primary fibroblast cells were subjected to Western blotting to detect endogenous TSPO. **(C)** Fibroblasts from carriers of wt and homozygous A147T and R162H mutations of TSPO were treated with cycloheximide (25 μg/ml) for the indicated times, and endogenous TSPO was then detected by specific rabbit anti-TSPO antibody. Equal amounts of protein were subjected to Western blot analyses, as determined by comparing the amount of beta 1 tubulin. **(D)** Densitometry results for endogenous TSPO after treatment with cycloheximide were quantified as percentage of the initial TSPO protein level (0h of CHX treatment) and normalized to the intensity of beta 1 tubulin, and then plotted. Data are shown as mean ± SEM from three independent experiments.

Next, we aimed to analyse the stability of native and mutant proteins in living cells using the cycloheximide (CHX) chase protein degradation assay. We chose primary dermal fibroblasts which were genotyped prior to assay performance. Primary dermal fibroblasts express robust amounts of TSPO which localises to mitochondria (Fig 5A), as verified by co-localisation with ATP synthase beta subunit (ATPB). A rabbit polyclonal affinity purified antibody, TSPO-MIL, which recognized an epitope comprising the last 14 amino acids of human TSPO, revealed a single 18 kDa band (Fig 5B) in western blots from fibroblast protein extracts. The specificity of the TSPO-MIL antibody was confirmed with peptide blocking experiments (S3 Fig). For the cycloheximide chase assay, we generated six primary dermal fibroblast cell lines from two healthy donors each carrying either wild-type or a homozygous genotype for the A147T or R162H TSPO variants. The TSPO protein stability was assessed by incubating cells for a maximum of 24 hours with 25 μg/ml of CHX, a well-known inhibitor of eukaryotic translation. The TSPO protein expression was quantified at different time points after CHX treatment as percentage of the initial TSPO protein level (0h of CHX treatment) (Fig 5C), and normalised to beta 1 tubulin (BTUB1) expression. Wild-type TSPO remained stable even after 24 hours (107±5%), whereas both mutant proteins showed significant protein degradation. The A147T mutant showed a tendency of degradation after 16 hours (93±9%), and the protein amount was further reduced to 75±5% of initial values after 24h, whereas R162H mutant protein levels were likewise reduced to 76±13% only at the final time point (Fig 5D). Results from protein degradation analysis suggest that higher structural flexibility of both mutant proteins is accompanied by decreased protein stability.

## Discussion

In this study, we conducted systematic bioinformatic analysis of the human TSPO gene with the aim to identify potential deleterious nsSNPs and their effect on protein structure and stability. SNPs are common genetic variants that occur at a frequency of approximately 1 in 300 bp throughout the human genome [25]. With the number of newly identified SNPs rapidly growing, reaching over 135 million validated SNPs in NCBI dbSNP database (build 150), there is a need to prioritize SNPs that are functionally deleterious from neutral SNPs [26]. The nsSNPs represent a small proportion of all SNPs which lead to amino acid change that could cause a loss of thermodynamic stability, as well as aberrant folding of mutated protein. Changes in amino acid properties could disrupt local environment by changing protein´s size, hydrophobic contacts, surface charge distribution and residue-solvent interactions [27], however if they occur at regions which are non-essential for the protein function they will be probably neutral. Therefore, numerous bioinformatics tools have been developed to predict the impact of amino acids substitution on protein structure and function. It is assumed that deleterious mutations are most likely to occur in evolutionary conserved regions [28], thus allowing us to identify amino acid residues that could be functionally important. In fact, amino acid residues which evolve under strong purifying selection are found to be most likely associated with human diseases [29]. We indeed identified two deleterious mutation clusters in the highly conserved regions in TMDs 2 and 5. These two adjacent transmembrane domains encompass a cholesterol recognition/interaction amino acid consensus sequence (CRAC) domain, which is essential for binding of cholesterol and other TSPO ligands. However, covariance analysis of over 2000 TSPO sequences [30] have identified robust association between helices 4 and 5, which is suggestive of their critical role for the stability and function of TSPO. Consequently, we expect that mutations in highly conserved regions in TMDs 2, 4, and 5 will have severe consequences on TSPO ligand binding and function.

From the all nsSNPs analysed, only A147T and R162H variants were present in human populations with a frequency of about 30%, whereas all the other nsSNPs were very rare (MAF<0.003). The effect of A147T mutation on pregnenolone synthesis, and on the protein structure has been demonstrated in previous studies [17, 22, 31, 32], however only one study so far investigated role of R162H mutation in mood disorder [33]. Nevertheless our computational analysis has shown that about 40 percent of all variants could have deleterious effect on protein structure and function. The high number of identified deleterious mutations could be explained by the evolutionary conservation of TSPO protein.

Since it has been shown that codon usage could compromise protein structure and function [19, 21, 34], we additionally analysed codon usage in the TSPO gene. We identified three polymorphisms located in a rare codon cluster between amino acids 110 and 124, which could affect timing of folding and insertion of TSPO into the membrane. As previously shown [21], exchange of the frequent codons with rare codons in the cluster of infrequently used codons could influence the translation rate, which in turn may potentially affect protein folding. Nevertheless, possible effects of TSPO sSNPs on protein folding need to be validated experimentally. Micro Scale Thermophoresis (MST) which is very sensitive to changes in protein structure and conformation or trypsin accessibility assay may possibly be performed, however for this type of experiments, proteins needs to be heterologously expressed.

While the physiological role of TSPO is still under investigation, it is used routinely as a biomarker for various neurodegenerative, neuroinflammatory and psychiatric disorders [35–37], although the binding of high affinity PET ligands has been shown to be dependent on the TSPO genotype, specifically the A147T variant [38]. Previous studies [22, 31] have demonstrated the structural impact of the A147T mutation in bacterial and in mouse system, however a number of discrepancies have been observed between structures of prokaryotic and mammalian TSPO homologues. TSPO from Rhodobacter sphaeroides has dimeric organisation, and substitution of A139T, which is equivalent to the human A147T mutation, resulted in conformational changes in LP1, CRAC domain, and TMD2. On the other side, the NMR structure of a A147T mouse TSPO showed contradictory results. The mouse TSPO has been shown to exist as a monomer, with the A147T mutation having only an effect on TMD1 and LP1, without alterations in CRAC domain. Furthermore recent data showed that mouse TSPO exist in dynamic monomer-dimer equilibrium in the membrane [39].

To study the impact of mutations at the structural level, we generated high quality structures of human native and mutant TSPO proteins by homology modelling using the *Bacillus cereus* structure as most suitable template. Even though we used the bacterial TSPO structure as a template for homology modelling, our results concerning the A147T mutation are more similar to the mouse model of A147T polymorph [31] then to the bacterial A139T [22], which has additional 7.70^o^ tilt of TMD2 towards TMD5, an orientation which could not be observed in our model. In a next step, we analyzed the impact of A147T and R162H mutations on the protein structure. Our MDS experiments were conducted over a period of 30 ns and showed that the A147T mutant presented higher flexibility in the loop regions when compared to the native protein. We suggest that, this increased structural flexibility possibly affects ligand binding. Our data indicate that the TSPO protein is highly dynamic in the absence of a ligand, which is reflected by high RMSD values, as expected for the naturally flexible protein like TSPO.

To test whether higher flexibility of the mutant protein could affect its stability, we estimated half-life kinetics of native and mutant proteins in human dermal fibroblasts, which were generated from two healthy donors each carrying a specific genotype. TSPO as a integral membrane protein has a relatively long half-life of 54.4±12 hours [40]. Nevertheless, using CHX as a potent translational inhibitor, we could show that both mutants have significantly shorter half-life kinetics suggestive of altered protein stability. It is of note that from the fairly long half-life of TSPO protein, we did not expect to see great differences in half-life kinetics between native and mutant proteins due to the limitations of CHX degradation assay which can be performed for a maximum of 24 hours. We can only speculate that a shortened half-life of the mutant A147T protein could explain its association with predisposition to various disorders [13–16] and reduced pregnenolone synthesis [17].

In summary, using a combined bioinformatics approach we identified several potentially deleterious SNPs which could affect the structure and stability of the TSPO protein. The structural consequences of A147T and R162H nsSNPs as predicted using MDS have shown a significantly higher flexibility of mutant structures, which was associated with a shortened half-life of mutant proteins. Our study provides further insights into the structural consequences of A147T mutation and the first characterisation of R162H variant for the human TSPO protein and may facilitate the virtual screening for novel TSPO ligands. Nevertheless, the three-dimensional crystal structures of the human TSPO proteins are still necessary for validation of structural changes suggested by our approach.

## Material and methods

### Dataset collection

SNPs of the human TSPO gene were retrieved from the National Center for Biotechnology Information (NCBI) SNPs database, (dbSNP, build 141) [41] and Exome Aggregation Consortium (ExAC) database, [42]. All redundant, outdated and SNPs in non-canonical transcripts were removed. The amino acid sequence of human TSPO (accession number: AAQ75703.1) was retrieved from the NCBI website.

### Computational methods for detection of deleterious mutations

For prediction of the effect of nsSNPs on the protein structure and stability we used eight computational algorithms. Protein Variation Effect Analyzer, (PROVEAN) [43], predicts the functional impact of protein sequence variations as "neutral" or "deleterious". The sorting intolerant from tolerant (SIFT) method [44], which is based on the degree of conservation of amino acids in sequence alignments, predicts whether an amino acid substitution affects protein function. SIFT assigns scores where a variant with a score less than 0.05 is considered deleterious, whereas a variant with a score greater than 0.05 is considered tolerated. Polymorphism Phenotyping v2 (PolyPhen-2) [45] is a tool which predicts possible impact of an amino acid substitution on the structure and function of a human protein using straightforward physical and comparative considerations. Mutations with probabilistic score bellow 0.15 are classified as benign, the ones in the range of 0.15 to 0.84 are classified as "possibly damaging", and mutation is classified as "most likely damaging" if the score is higher than 0.85. Predictor of human Deleterious Single Nucleotide Polymorphisms (PhD-SNP) [46] is a Support Vector Machine (SVM) single sequence based method which predicts whether a nSNP is a neutral polymorphism or a disease associated polymorphism. Screening for Non-Acceptable Polymorphisms 2 (SNAP2) [47] method utilizes sequence information, secondary structure and residue conservation as well as neural networks to predict whether the mutation is neutral or non-neutral. SNPs&GO [48] is SVM based method which predicts if a given single point protein variation can be classified as disease associated or neutral using evolutionary information, and functions encoded in gene ontology terms. Mutations with a score greater than 0.5 are classified as disease related. Functional Analysis through Hidden Markov Models (FATHMM) [49] predicts the functional effects of protein missense mutations as tolerated or damaging, by combining sequence conservation within hidden Markov models (HMMs). I-Mutant 3 [50] calculates the thermodynamic stability change between wild type and mutant proteins. A single amino acid substitution can result in a significant change in protein stability, which is denoted by the protein stability free energy change (DDG) value. I-Mutant 3 predictions are classified into three classes: destabilizing mutations (DDG<-0.5 Kcal/mol), stabilizing mutations (DDG>0.5 Kcal/mol) and neutral mutations (-0.5< = DDG< = 0.5 Kcal/mol). We additionally estimated the evolutionary conservation rate of each residue in TSPO using ConSurf [51], which quantifies the degree of conservation for each amino acid position in a given alignment. The extent of conservation of each residue is presented as a score in the range of 1–9, with 1 denotes highly variable sites, and 9 evolutionary conserved sites.

Effect of synonymous mutations on protein structure and function were probed using codon usage analyser [20]. The codon quality of coding sequences can be represented as codon usage frequency or as relative adaptiveness values. In contrast to the codon usage frequency the relative adaptiveness takes into account the number of codons which code for the respective amino acid, and is obtained by setting the codon with the highest frequency value to 100%, and all other codons for the same codon are scaled accordingly.

### Homology modeling of the human TSPO structures

Homology model of human TSPO was built using YASARA molecular modelling program version 16.4.6 [52]. The hm_build.mcr macro of the YASARA software was used with the default parameters except the maximum oligomerization state was set to one. The amino acid sequence of TSPO (accession number: AAQ75703.1) was submitted to modeling, and template structures were searched by running six iterations of PSI-BLAST. For the top scoring templates (PDB ID: 4UC1, 4RYO, and 4UC3) from the protein data bank a total of 6 models were created, and the quality of the model structure was evaluated by considering the overall Z-score. The Z-score has been defined as the weighted averages of the individual Z-scores using the formula, Z-score = 0.1456 x Dihedrals + 0.3906 x Packing1D + 0.4656 x Packing3D [52]. TSPO model structure with the Z-score of -0.465 which was based on Bacillus cereus TSPO structure (PDB ID: 4RYO solved at 1.6 Å) was subjected to further refinement using the md_refine.mcr macro of YASARA, while the models with lower quality Z-scores were discarded. A147T and R162H mutant structures were obtained by mutating corresponding positions in the TSPO model structure. After the mutation, the structures were subjected to an energy minimization with the YAMBER force field as described previously [53]. The structures of wt and mutant TSPO proteins were superimposed and aligned using MUSTANG program implemented in the YASARA software package [54]. TSPO models created in this study are made available on institute homepage:

(http://www.uni-regensburg.de/medizin/psychiatrie-psychotherapie/forschung/molekulare-neurowissenschaften/data/index.html).

### Molecular dynamics simulation

Molecular dynamics (MD) simulation technique was used in order to understand the effect of point mutations on TSPO protein structure and stability. The energy minimised structures of the native and mutant TSPO proteins were used as starting points for the MD simulations, which were carried out with YASARA software using the md_runmembrane.mcr macro and the AMBER04 force field.

The TSPO simulation was set up automatically by first scanning the protein for exposed transmembrane helices. Based on the identified secondary structure elements, the protein was oriented normally with respect to the plane of the membrane and the XZ-plane. The equilibrated membrane, consisting of phosphatidylethanolamine molecules was enclosed in the simulation cell of size [69x98x65] Å and the native or mutant proteins were then embedded into the membrane. The simulation cell contained around 22 000 atoms which included the TSPO protein together with phosphatidylethanolamine and water molecules. During the equilibration phase which lasted 250 ps, the membrane was artificially stabilized to avoid distortions while the simulation cell adapted to the pressure exerted by the membrane. The MD was run for 30–35 ns at constant temperature of 298K, and pressure with a time step of 2 fs and snapshots were saved at every 25 ps, as defined in md_runmembrane.mcr macro.

The trajectory files generated by MD simulations were analysed using YASARA to obtain the root-mean-square deviation (RMSD), root-mean-square-fluctuation (RMSF), and radius of gyration (Rg) values, which were statistically analysed to determine the differences in the RMSD, RMSF, and Rg values between native and mutant protein structures.

### Study approval

The present study has obtained approval of the ethics review board of the University of Regensburg, Germany (Reference No. 13-101-0271) and has been performed in accordance with the ethical standards laid down in the 1964 Declaration of Helsinki and its later amendments. Participants were recruited from March 09, 2015 to September 19, 2016, from students, staff, and employees of University of Regensburg. All the participants were healthy, free from any medication and skin allergy. Informed consent was given by each proband participating in the study.

### Cell lines and culture conditions

Adult human dermal primary fibroblasts were derived from the skin biopsies of different donors harbouring either wild-type genotype or, homozygous genotype for A147T, or R162H TSPO mutations. Healthy skin (4 mm) was obtained according to standard protocols from the Department of Dermatology, University Hospital Regensburg, Germany. Dermal tissue was cut into 0.5-mm pieces and subsequently seeded in 6 well cell culture dishes in fibroblast growth medium (DMEM high-glucose with 4.5 g/l L-glutamine, supplemented with 10% fetal calf serum, 100 U/ml penicillin and 0.1 mg/ml streptomycin, Thermo Fischer Scientific, Darmstadt, Germany). After 10–15 days, an outgrowth of single fibroblasts was observed. Cultures were maintained in humidified air (5% CO_2_) at 37^o^ C, and medium was changed three times a week. After reaching 80% confluency, cells were detached using trypsin–EDTA (Thermo Fischer Scientific, Darmstadt, Germany) and subcultured in 75-cm^2^ cell culture flasks with fibroblast growth medium.

### Genotyping of rs6791 and rs6792 SNPs in TSPO

Genomic DNA was extracted from 4 ml of whole blood with QIAamp DNA blood maxi kit (Qiagen, Hilden, Germany) according to the manufacturer’s protocol. DNA quality was assessed utilizing optical absorbance and gel electrophoresis. Exon 4 of the TSPO gene containing the polymorphisms rs6971 (Ala or Thr at position 147), and rs6972 (Arg or His at position 162), as well as protein coding exons 2 and 3 and their respective exon/intron junctions were PCR amplified and sequenced using Sanger method. Sequencing primers used in this study are listed in S4 Table. Sequencing data were analysed using SnapGene software (from GSL Biotech; available at snapgene.com).

### Western blot and TSPO antibody generation

Whole cell protein samples were sonicated and boiled in RIPA buffer and total protein was quantified using a micro-BCA colorimetric assay (Pierce, Thermo Fischer Scientific). Protein samples were separated by SDS-polyacrylamide gel electrophoresis on 15% gels and subsequently transferred onto Immobilon®-P PVDF membrane (Millipore, Bedford, MA, USA). Incubation of primary and secondary antibodies was carried out at 4°C ON, respectively.

The peptide RDNHGWRGGRRLPE containing amino acids 156–169 of human TSPO was used to generate affinity-purified rabbit polyclonal antibody (Davids Biotech, Regensburg, Germany). Specificity of this antibody termed anti TSPO-MIL was tested by western blotting, and immunostaining (S3 Fig). Western blot and immunocytochemistry were performed using either plain primary antibody diluted 1:5000 for western blotting and 1:500 for immunocytochemistry or primary antibody incubated with 1 μg/ml of blocking peptide for 1 hour. ATP synthase beta subunit mouse monoclonal antibody (ab14730, Abcam, Cambridge, UK) was used in 1:1000 dilution for specific mitochondrial staining.

### Cycloheximide protein degradation assay and quantitative Western blot analysis

The turnover of proteins was determined using cycloheximide inhibition of protein de novo synthesis. Cells were seeded into 6 well plates, and subsequently treated with 25 μg/ml cycloheximide (Sigma, Taufkirchen, Germany) for the indicated time points.

Proteins were separated by SDS-PAGE and blotted onto Immobilon®-FL membrane (LI-COR Bioscience, Bad Homburg, Germany). TSPO protein was detected using the TSPO-MIL antibody, multiplexed with beta 1 tubulin antibody (clone E7, Developmental Studies Hybridoma Bank, Iowa, USA) which was used as a loading control. IRDye 700 and 800 infrared secondary antibodies were used for simultaneous detection using the Odyssey Infrared imaging system (LI-COR Bioscience, Bad Homburg, Germany). TSPO band densities were quantified with Odyssey Image Studio v4.0 software as percentage of the initial TSPO protein level (0h of CHX treatment) and normalized against beta 1 tubulin signal from the same blot.

### Statistical analysis

Statistical analysis were performed with Sigma Plot 12 (Systat Software, San Jose, USA), and Igor Professional 6.37 (Wavemetrics, Portland, USA). Data were tested for normal distribution prior to the statistical analysis. Parametric data were tested with Student’s t-test and nonparametric data with the Wilcoxon signed-rank test. Level of significance was set at p < 0.05. All data were presented as mean ± SEM.

## Supporting information

S1 Fig ConSurf conserved regions. The ConSurf algorithm at consurf.tau.ac.il. was used to provide conservation score for the amino acids of human TSPO. Interestingly, 11 out of 21 of the predicted deleterious mutations occur in highly conserved regions of TSPO, depicted in dark violet colour. Remarkably, all of them but 2 cluster in 2 conserved amino acid stretches in TMD2 (aa 44–65) and in TMD5 (aa 133–150), which are facing each other.
(TIF)

S2 Fig Codon usage analysis of human TSPO gene. Relative adaptiveness values for wt TSPO codons are shown in black, whereas three sSNPs with strongest effect on codon usage are depicted in red. The SNPs at positions 113 and 114 which are located in a rare codon usage cluster are showing large reduction of relative adaptiveness values from 100 to 18.
(TIF)

S3 Fig Confirmation of antibody specificity by peptide blocking. Western blot and immunocytochemistry were performed using either plain primary antibody or primary antibody incubated with 1 μg/ml of peptide which was used to generate the anti-TSPO antibody. **(A)** Western blot analyses of protein extracts from HEK-293 cells and fibroblasts revealed single bands corresponding to the molecular size of TSPO, which disappeared after incubation with blocking peptide, thus confirming antibody specificity. Beta 1 tubulin antibody was used as loading control. **(B)** Similar results were obtained using immunocytochemistry, where co-localisation of TSPO with ATPB confirmed mitochondrial localisation of TSPO. Scale bar 10 μm.
(TIF)

S1 Table List of all TSPO SNPs. (DOCX)

S2 Table ConSurf table, conservation score. (DOCX)

S3 Table Codon usage analysis results. (DOCX)

S4 Table Primers used for sequencing. (DOCX)
