# Supplementary figures and images for: Effects of genetic variants in the TSPO gene on protein structure and stability
Source: PLoS One. 2018 Apr 11;13(4):e0195627. doi: 10.1371/journal.pone.0195627 (PMC5895031; doi:10.1371/journal.pone.0195627)

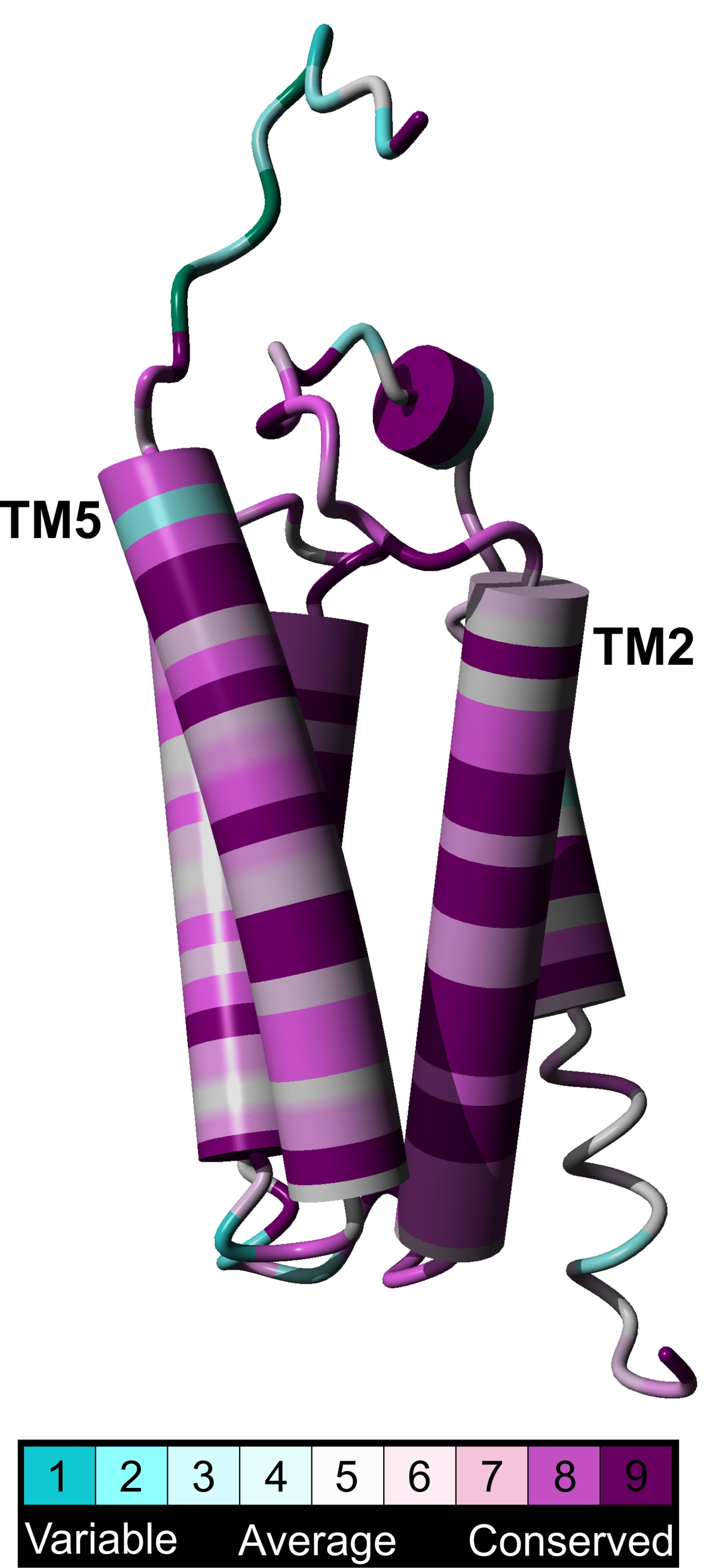

Supplement: S1 Fig — The ConSurf algorithm at consurf.tau.ac.il. was used to provide conservation score for the amino acids of human TSPO. Interestingly, 11 out of 21 of the predicted deleterious mutations occur in highly conserved regions of TSPO, depicted in dark violet colour. Remarkably, all of them but 2 cluster in 2 conserved amino acid stretches in TMD2 (aa 44–65) and in TMD5 (aa 133–150), which are facing each other. (TIF) [file pone.0195627.s001.tif]

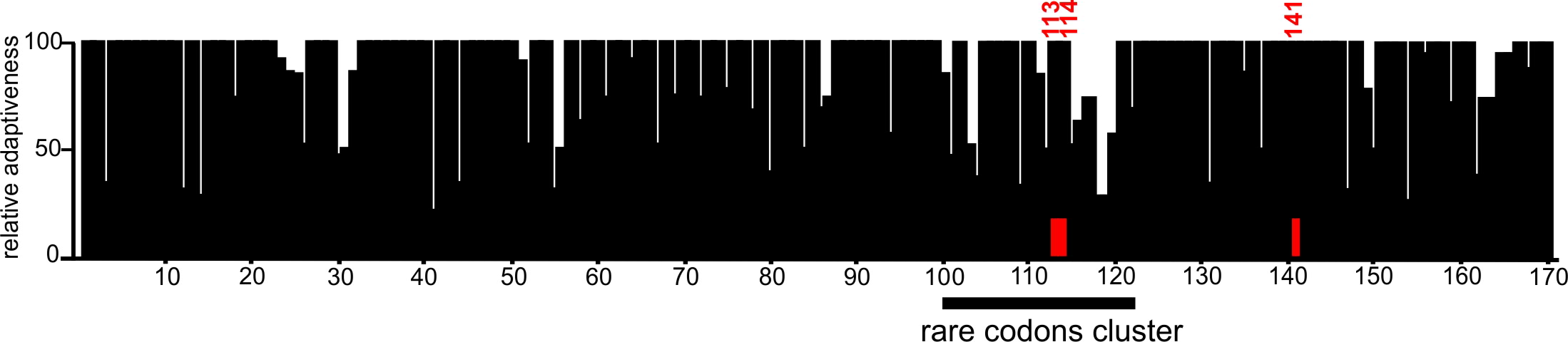

Supplement: S2 Fig — Relative adaptiveness values for wt TSPO codons are shown in black, whereas three sSNPs with strongest effect on codon usage are depicted in red. The SNPs at positions 113 and 114 which are located in a rare codon usage cluster are showing large reduction of relative adaptiveness values from 100 to 18. (TIF) [file pone.0195627.s002.tif]

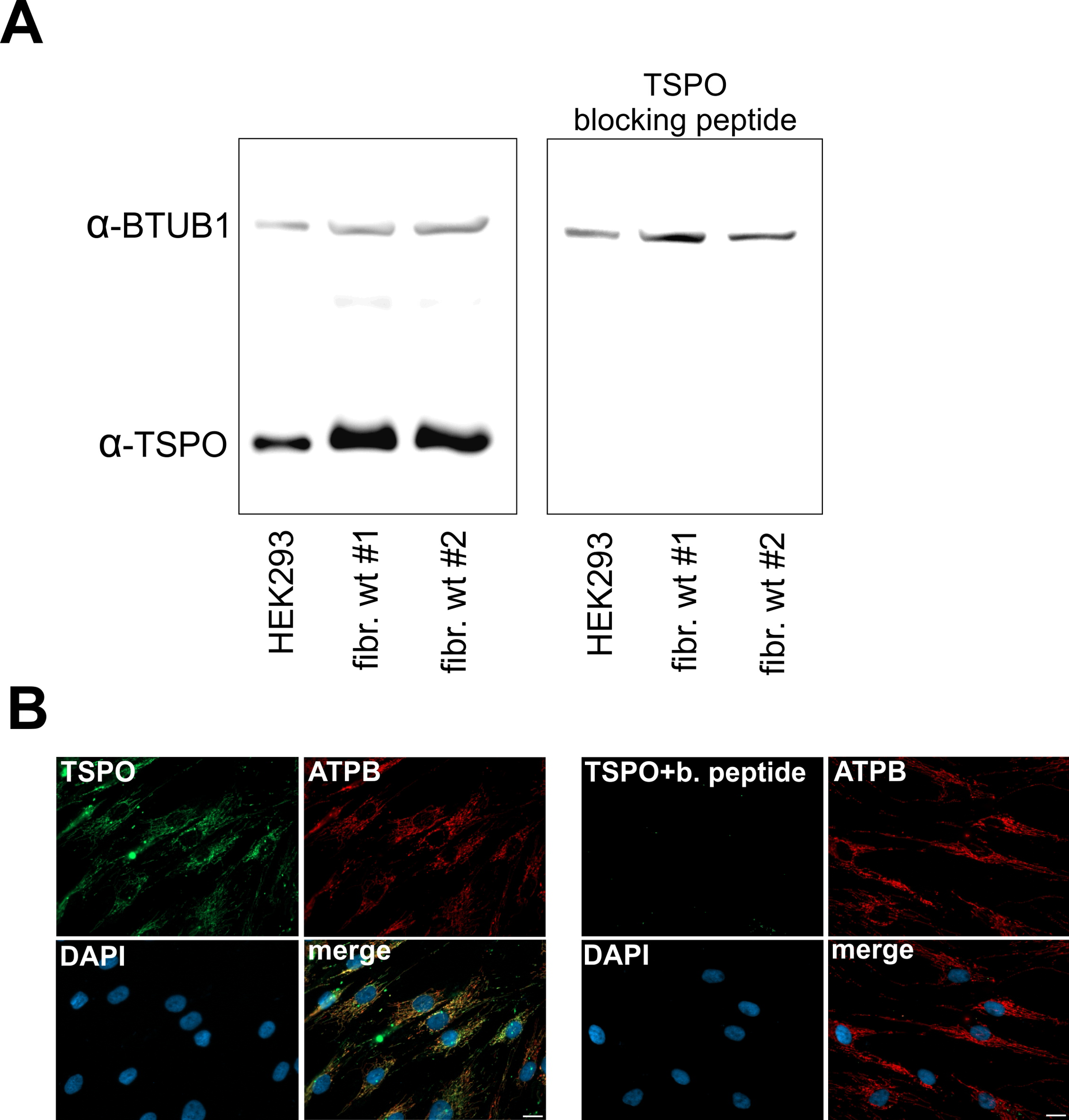

Supplement: S3 Fig — Western blot and immunocytochemistry were performed using either plain primary antibody or primary antibody incubated with 1 μg/ml of peptide which was used to generate the anti-TSPO antibody. (A) Western blot analyses of protein extracts from HEK-293 cells and fibroblasts revealed single bands corresponding to the molecular size of TSPO, which disappeared after incubation with blocking peptide, thus confirming antibody specificity. Beta 1 tubulin antibody was used as loading control. (B) Similar results were obtained using immunocytochemistry, where co-localisation of TSPO with ATPB confirmed mitochondrial localisation of TSPO. Scale bar 10 μm. (TIF) [file pone.0195627.s003.tif]
