# Supplementary material for: Effects of genetic variants in the TSPO gene on protein structure and stability
Source: PLoS One. 2018 Apr 11;13(4):e0195627. doi: 10.1371/journal.pone.0195627 (PMC5895031; doi:10.1371/journal.pone.0195627)
Supplement: S1 Table — (DOCX) [file pone.0195627.s004.docx]

**Supplementary Table 1.** List of all TSPO SNPs used in this study

| # | SNP ID | Amino acid position | Mutation | Type | Allele  frequency | |
| --- | --- | --- | --- | --- | --- | --- |
| 1 | rs138908 | - | G/T | 5´SNPs |  |  |
| 2 | rs9333315 | - | C/T | 5´SNPs |  |  |
| 3 | rs9333316 | - | C/T | 5´SNPs |  |  |
| 4 | rs34205127 | - | -/G | 5´SNPs |  |  |
| 5 | rs113536963 | - | A/C | 5´SNPs |  |  |
| 6 | rs115144779 | - | C/T | 5´SNPs |  |  |
| 7 | rs182381541 | - | A/G | 5´SNPs |  |  |
| 8 | rs192794013 | - | C/G | 5´SNPs |  |  |
| 9 | rs529386791 | - | A/G | 5´SNPs |  |  |
| 10 | rs532718233 | - | G/T | 5´SNPs |  |  |
| 11 | rs539748252 | - | A/G | 5´SNPs |  |  |
| 12 | rs552628902 | - | C/T | 5´SNPs |  |  |
| 13 | rs558045908 | - | C/G | 5´SNPs |  |  |
| 14 | rs564404579 | - | C/T | 5´SNPs |  |  |
| 15 | rs566091388 | - | A/G | 5´SNPs |  |  |
| 16 | rs568501993 | - | C/T | 5´SNPs |  |  |
| 17 | rs760878391 | - | A/G | 5´SNPs |  |  |
| 18 | rs769914892 | - | A/C | 5´SNPs |  |  |
| 19 | rs770880317 | - | C/T | 5´SNPs |  |  |
| 20 | rs771942072 | - | C/G | 5´SNPs |  |  |
| 21 | rs774238168 | - | C/T | 5´SNPs |  |  |
| 22 | rs777916643 | - | A/G | 5´SNPs |  |  |
| 23 | rs764229144 | A2D | A/C | nsSNPs | 0.00008179 |  |
| 24 | rs753849621 | W5R | C/T | nsSNPs | 0.00001713 |  |
| 25 | rs765728030 | A8T | A/G | nsSNPs | 0.0001348 |  |
| 26 | rs567132675 | G10S | A/G | nsSNPs | 0.00006363 |  |
| 27 | rs758845609 | L13P | C/T | nsSNPs | 0.00006265 |  |
| 28 | rs187866832 | A14V | C/T | nsSNPs | 0.003467 |  |
| 29 | rs777431441 | V21L | C/G | nsSNPs | 0.00006143 |  |
| 30 | rs748778969 | G22A | C/G | nsSNPs | 0.00000929 |  |
| 31 | rs778946143 | G28S | A/G | nsSNPs | 0.00001037 |  |
| 32 | rs775263818 | G30D | A/G | nsSNPs | 0.00006101 |  |
| 33 | rs566580110 | R32C | C/T | nsSNPs | 0.00006118 |  |
| 34 | rs750994845 | S41W | C/G | nsSNPs | 0.00006466 |  |
| 35 | rs550303992 | P44L | C/T | nsSNPs | 0.00006706 |  |
| 36 | rs566547284 | G63S | A/G | nsSNPs | 0.000008353 |  |
| 37 | rs139234976 | G63D | A/G | nsSNPs | 0.000008347 |  |
| 38 | rs756858058 | Y65F | A/T | nsSNPs | 0.000008311 |  |
| 39 | rs199899658 | E70D | C/G | nsSNPs | 0.0004797 |  |
| 40 | rs749849080 | T75I | C/T | nsSNPs | 0.000008259 |  |
| 41 | rs372235648 | A78V | C/T | nsSNPs | 0.000008256 |  |
| 42 | rs746919529 | G83R | C/G | nsSNPs | 0.00002476 |  |
| 43 | rs754824182 | L84F | C/T | nsSNPs | 0.000008255 |  |
| 44 | rs142445069 | A94V | C/T | nsSNPs | 0.0006368 |  |
| 45 | rs775043588 | P97L | C/T | nsSNPs | 0.000008277 |  |
| 46 | rs760110771 | A102T | A/G | nsSNPs | 0.000008313 |  |
| 47 | rs775654599 | R103Q | A/G | nsSNPs | 0.00002499 |  |
| 48 | rs761543515 | Q104E | C/G | nsSNPs | 0.000008338 |  |
| 49 | rs143915407 | G106V | G/T | nsSNPs | 0.00005853 |  |
| 50 | rs752645452 | V115I | A/G | nsSNPs | 0.00003266 |  |
| 51 | rs148614502 | A119V | C/T | nsSNPs | 0.001154 |  |
| 52 | rs200880548 | A120P | C/G | nsSNPs | 0.00003 |  |
| 53 | rs779150979 | A120G | C/G | nsSNPs | 0.00002997 |  |
| 54 | rs780467525 | V124M | A/G | nsSNPs | 0.00003 |  |
| 55 | rs373738253 | P131L | C/T | nsSNPs | 0.00005943 |  |
| 56 | rs773881998 | A133T | A/G | nsSNPs | 0.00003 |  |
| 57 | rs767027529 | A134T | A/G | nsSNPs | 0.00002913 |  |
| 58 | rs775344095 | R135C | C/T | nsSNPs | 0.0000552 |  |
| 59 | rs760654235 | R135H | A/G | nsSNPs | 0.0000274 |  |
| 60 | rs757578697 | P139T | A/C/T | nsSNPs | 0.00005058 |  |
| 61 | n/a | P139S | C/T | nsSNPs | 0.00002529 |  |
| **62** | **rs6971** | **A147T** | **C/T** | **nsSNPs** | **0.2237** |  |
| 63 | rs141002863 | A147M | C/T | nsSNPs | 0.0000442 |  |
| 64 | rs774036527 | L150F | C/T | nsSNPs | 0.00005937 |  |
| 65 | rs771545866 | V154I | A/G | nsSNPs | 0.00003161 |  |
| 66 | rs775001391 | R156W | C/T | nsSNPs | 0.0000164 |  |
| 67 | rs375211541 | R156Q | A/G | nsSNPs | 0.00006544 |  |
| **68** | **rs6972** | **R162H** | **A/G** | **nsSNPs** | **0.3029** |  |
| 69 | rs776603192 | R165W | C/T | nsSNPs | 0.00009964 |  |
| 70 | rs761740678 | R165Q | A/G | nsSNPs | 0.00004981 |  |
| 71 | rs765102690 | R166W | C/T | nsSNPs | 0.00002546 |  |
| 72 | rs8192467 | R166L | G/T | nsSNPs | 0.0005365 |  |
| 73 | rs9333342 | E169Q | C/G | nsSNPs | 0.0001055 |  |
| 74 | rs568411305 | E169V | A/T | nsSNPs | 0.0002 |  |
| 75 | rs9333332 | L31 | C/T | sSNPs | 0.00011839 |  |
| 76 | rs9333341 | L137 | C/G | sSNPs | 0.00004152 |  |
| 77 | rs142042960 | L141 | G/A | sSNPs | 0.00007467 |  |
| 78 | rs192201237 | T55 | G/A | sSNPs | 0.00027816 |  |
| 79 | rs372615034 | A14 | G/A | sSNPs | 0.00002635 |  |
| 80 | rs375039721 | A35 | C/T | sSNPs | 0.00002795 |  |
| 81 | rs376234950 | Q88 | G/A | sSNPs | 0.00001647 |  |
| 82 | rs376383649 | G28 | A/C/T | sSNPs | 0.00002159 |  |
| 83 | rs377098874 | Y85 | C/T | sSNPs | 0.00004118 |  |
| 84 | rs745517102 | C153 | C/T | sSNPs | 0.00004146 |  |
| 85 | rs745897744 | A120 | A/C | sSNPs | 0.00000840 |  |
| 86 | rs747473912 | F146 | C/T | sSNPs | 0.00002490 |  |
| 87 | rs747530187 | A125 | C/G/T | sSNPs | 0.00000836 |  |
| 88 | rs748472790 | A147 | G/A | sSNPs | 0.00001658 |  |
| 89 | rs749824230 | V26 | C/T | sSNPs | n/a |  |
| 90 | rs750349204 | P15 | C/T | sSNPs | n/a |  |
| 91 | rs751910716 | P44 | G/A | sSNPs | 0.00004160 |  |
| 92 | rs752041846 | A133 | C/T | sSNPs | 0.00002507 |  |
| 93 | rs753588433 | S64 | C/T | sSNPs | 0.00002471 |  |
| 94 | rs754183202 | A119 | G/A | sSNPs | 0.00003401 |  |
| 95 | rs755760336 | F20 | C/T | sSNPs | 0.00003686 |  |
| 96 | rs755909339 | A118 | G/A | sSNPs | 0.00002552 |  |
| 97 | rs758354864 | K77 | G/A | sSNPs | 0.00001647 |  |
| 98 | rs758458209 | L144 | C/T | sSNPs | 0.00001659 |  |
| 99 | rs759540587 | L113 | A/G | sSNPs | 0.00000834 |  |
| 100 | rs760894949 | G30 | C/T | sSNPs | 0.00002266 |  |
| 101 | rs761915112 | Y34 | C/T | sSNPs | 0.00001366 |  |
| 102 | rs762240782 | P7 | C/T | sSNPs | 0.00000857 |  |
| 103 | rs762605393 | V129 | G/A | sSNPs | 0.00000839 |  |
| 104 | rs763364246 | S41 | G/A | sSNPs | 0.00003791 |  |
| 105 | rs763988323 | R135 | C/G | sSNPs | 0.00002494 |  |
| 106 | rs765876557 | L109 | G/A | sSNPs | 0.00000832 |  |
| 107 | rs767313988 | L114 | G/A | sSNPs | 0.00000835 |  |
| 108 | rs770108373 | T148 | C/T | sSNPs | 0.00000828 |  |
| 109 | rs770801880 | S23 | C/G | sSNPs | 0.00000947 |  |
| 110 | rs771101046 | P96 | C/A | sSNPs | 0.00000824 |  |
| 111 | rs772665846 | T123 | C/T | sSNPs | 0.00000837 |  |
| 112 | rs774883859 | Y62 | C/T | sSNPs | 0.00005765 |  |
| 113 | rs776912609 | Y127 | C/T | sSNPs | 0.00000838 |  |
| 114 | rs780169964 | A145 | C/T | sSNPs | 0.00001659 |  |
| 115 | rs6973 | - | G/T | 3´SNPs |  |  |
| 116 | rs8192468 | - | G/T | 3´SNPs |  |  |
| 117 | rs79307767 | - | A/G | 3´SNPs |  |  |
| 118 | rs111607654 | - | G/T | 3´SNPs |  |  |
| 119 | rs112069650 | - | C/T | 3´SNPs |  |  |
| 120 | rs150429499 | - | C/T | 3´SNPs |  |  |
| 121 | rs372430939 | - | C/T | 3´SNPs |  |  |
| 122 | rs373324032 | - | C/G | 3´SNPs |  |  |
| 123 | rs377258040 | - | C/G | 3´SNPs |  |  |
| 124 | rs536692143 | - | A/G | 3´SNPs |  |  |
| 125 | rs542134891 | - | A/G | 3´SNPs |  |  |
| 126 | rs546066736 | - | A/C | 3´SNPs |  |  |
| 127 | rs547679650 | - | C/T | 3´SNPs |  |  |
| 128 | rs554144519 | - | A/T | 3´SNPs |  |  |
| 129 | rs557866422 | - | A/G | 3´SNPs |  |  |
| 130 | rs572631159 | - | A/G | 3´SNPs |  |  |
| 131 | rs745509989 | - | C/T | 3´SNPs |  |  |
| 132 | rs746491832 | - | A/G | 3´SNPs |  |  |
| 133 | rs755509592 | - | A/G | 3´SNPs |  |  |
| 134 | rs756260407 | - | A/G | 3´SNPs |  |  |
| 135 | rs756525698 | - | C/T | 3´SNPs |  |  |
| 136 | rs761830374 | - | C/T | 3´SNPs |  |  |
| 137 | rs763105738 | - | C/T | 3´SNPs |  |  |
| 138 | rs766380586 | - | C/G | 3´SNPs |  |  |
| 139 | rs769779822 | - | A/G | 3´SNPs |  |  |
| 140 | rs774887772 | - | C/T | 3´SNPs |  |  |
| 141 | rs775460412 | - | -/A | 3´SNPs |  |  |
| 142 | rs776758091 | - | A/G | 3´SNPs |  |  |
| 143 | rs778104603 | - | A/G | 3´SNPs |  |  |
| 144 | rs778153413 | - | C/G | 3´SNPs |  |  |
| 145 | rs105585 | - | A/G | intronic |  |  |
| 146 | rs138908 | - | G/T | intronic |  |  |
| 147 | rs138909 | - | A/T | intronic |  |  |
| 148 | rs138910 | - | C/T | intronic |  |  |
| 149 | rs138911 | - | G/T | intronic |  |  |
| 150 | rs138912 | - | C/G | intronic |  |  |
| 151 | rs138913 | - | A/G | intronic |  |  |
| 152 | rs138914 | - | A/G | intronic |  |  |
| 153 | rs138915 | - | A/G | intronic |  |  |
| 154 | rs138916 | - | C/T | intronic |  |  |
| 155 | rs138917 | - | G/T | intronic |  |  |
| 156 | rs762959 | - | C/T | intronic |  |  |
| 157 | rs762960 | - | C/T | intronic |  |  |
| 158 | rs2016151 | - | A/G | intronic |  |  |
| 159 | rs2157248 | - | A/G | intronic |  |  |
| 160 | rs2284099 | - | C/T | intronic |  |  |
| 161 | rs3937387 | - | A/C/G | intronic |  |  |
| 162 | rs5759195 | - | C/G | intronic |  |  |
| 163 | rs5759197 | - | C/T | intronic |  |  |
| 164 | rs5759198 | - | A/G | intronic |  |  |
| 165 | rs5759199 | - | A/G | intronic |  |  |
| 166 | rs5996280 | - | A/G | intronic |  |  |
| 167 | rs6003077 | - | A/T | intronic |  |  |
| 168 | rs6003078 | - | A/G | intronic |  |  |
| 169 | rs6003079 | - | C/G | intronic |  |  |
| 170 | rs6003080 | - | A/G | intronic |  |  |
| 171 | rs7287485 | - | A/G | intronic |  |  |
| 172 | rs8135638 | - | G/T | intronic |  |  |
| 173 | rs8141880 | - | A/C | intronic |  |  |
| 174 | rs9306372 | - | C/T | intronic |  |  |
| 175 | rs9333315 | - | C/T | intronic |  |  |
| 176 | rs9333316 | - | C/T | intronic |  |  |
| 177 | rs9333317 | - | C/T | intronic |  |  |
| 178 | rs9333318 | - | C/T | intronic |  |  |
| 179 | rs9333319 | - | -/G/GG | intronic |  |  |
| 180 | rs9333320 | - | G/T | intronic |  |  |
| 181 | rs9333321 | - | C/T | intronic |  |  |
| 182 | rs9333322 | - | C/G | intronic |  |  |
| 183 | rs9333323 | - | G/T | intronic |  |  |
| 184 | rs9333324 | - | A/G | intronic |  |  |
| 185 | rs9333325 | - | C/T | intronic |  |  |
| 186 | rs9333326 | - | A/G | intronic |  |  |
| 187 | rs9333327 | - | C/G | intronic |  |  |
| 188 | rs9333328 | - | A/G | intronic |  |  |
| 189 | rs9333329 | - | C/T | intronic |  |  |
| 190 | rs9333330 | - | A/G | intronic |  |  |
| 191 | rs9333331 | - | A/G | intronic |  |  |
| 192 | rs9333332 | - | C/T | intronic |  |  |
| 193 | rs9333333 | - | C/T | intronic |  |  |
| 194 | rs9333334 | - | A/C | intronic |  |  |
| 195 | rs9333335 | - | A/G | intronic |  |  |
| 196 | rs9333336 | - | C/G | intronic |  |  |
| 197 | rs9333337 | - | G/T | intronic |  |  |
| 198 | rs9333338 | - | C/T | intronic |  |  |
| 199 | rs9333339 | - | A/C | intronic |  |  |
| 200 | rs9333340 | - | -/GA | intronic |  |  |
| 201 | rs9623760 | - | A/G | intronic |  |  |
| 202 | rs9623761 | - | G/T | intronic |  |  |
| 203 | rs12166478 | - | C/T | intronic |  |  |
| 204 | rs12166481 | - | C/T | intronic |  |  |
| 205 | rs12166820 | - | C/T | intronic |  |  |
| 206 | rs12166896 | - | A/G | intronic |  |  |
| 207 | rs12185872 | - | A/T | intronic |  |  |
| 208 | rs12185873 | - | A/G | intronic |  |  |
| 209 | rs13058362 | - | C/T | intronic |  |  |
| 210 | rs13058657 | - | A/G | intronic |  |  |
| 211 | rs17003528 | - | A/G | intronic |  |  |
| 212 | rs34120993 | - | -/T | intronic |  |  |
| 213 | rs34205127 | - | -/G | intronic |  |  |
| 214 | rs34350979 | - | G/T | intronic |  |  |
| 215 | rs34474335 | - | A/G | intronic |  |  |
| 216 | rs34936584 | - | -/T | intronic |  |  |
| 217 | rs34963883 | - | -/A | intronic |  |  |
| 218 | rs35075244 | - | A/G | intronic |  |  |
| 219 | rs35991523 | - | -/A | intronic |  |  |
| 220 | rs56135426 | - | C/T | intronic |  |  |
| 221 | rs58430667 | - | A/C | intronic |  |  |
| 222 | rs59161843 | - | -/GTGA | intronic |  |  |
| 223 | rs60422492 | - | -/AG | intronic |  |  |
| 224 | rs60538423 | - | -/CGGGGCGGGGCAGG | intronic |  |  |
| 225 | rs60648790 | - | -/CGGG | intronic |  |  |
| 226 | rs60687062 | - | C/T | intronic |  |  |
| 227 | rs60859959 | - | -/GGGCGGGGCAGGAGGGAG | intronic |  |  |
| 228 | rs62232061 | - | C/T | intronic |  |  |
| 229 | rs62232062 | - | G/T | intronic |  |  |
| 230 | rs62232063 | - | C/T | intronic |  |  |
| 231 | rs62232064 | - | A/G | intronic |  |  |
| 232 | rs62234272 | - | A/G | intronic |  |  |
| 233 | rs62234273 | - | A/G | intronic |  |  |
| 234 | rs62234274 | - | C/T | intronic |  |  |
| 235 | rs71329181 | - | C/T | intronic |  |  |
| 236 | rs72619547 | - | A/G | intronic |  |  |
| 237 | rs73179066 | - | C/T | intronic |  |  |
| 238 | rs73418009 | - | C/T | intronic |  |  |
| 239 | rs73418011 | - | C/G | intronic |  |  |
| 240 | rs73418033 | - | C/T | intronic |  |  |
| 241 | rs73418038 | - | C/G | intronic |  |  |
| 242 | rs73418042 | - | C/T | intronic |  |  |
| 243 | rs73418045 | - | A/G | intronic |  |  |
| 244 | rs73886412 | - | C/T | intronic |  |  |
| 245 | rs73886413 | - | A/G | intronic |  |  |
| 246 | rs75034896 | - | A/C/G | intronic |  |  |
| 247 | rs76824102 | - | A/C | intronic |  |  |
| 248 | rs78387116 | - | A/T | intronic |  |  |
| 249 | rs78948972 | - | C/T | intronic |  |  |
| 250 | rs79641860 | - | A/C | intronic |  |  |
| 251 | rs79775338 | - | A/C | intronic |  |  |
| 252 | rs111353437 | - | C/G | intronic |  |  |
| 253 | rs111591296 | - | A/G | intronic |  |  |
| 254 | rs111879936 | - | -/T | intronic |  |  |
| 255 | rs112258901 | - | C/T | intronic |  |  |
| 256 | rs112311338 | - | G/T | intronic |  |  |
| 257 | rs112658712 | - | A/G | intronic |  |  |
| 258 | rs113013284 | - | C/T | intronic |  |  |
| 259 | rs113136237 | - | C/T | intronic |  |  |
| 260 | rs113313905 | - | C/G | intronic |  |  |
| 261 | rs113536963 | - | A/C | intronic |  |  |
| 262 | rs114150986 | - | A/G | intronic |  |  |
| 263 | rs114219847 | - | A/G | intronic |  |  |
| 264 | rs114618346 | - | A/G | intronic |  |  |
| 265 | rs115144779 | - | C/T | intronic |  |  |
| 266 | rs115708418 | - | G/T | intronic |  |  |
| 267 | rs116906704 | - | C/T | intronic |  |  |
| 268 | rs117202102 | - | C/T | intronic |  |  |
| 269 | rs117321410 | - | C/G | intronic |  |  |
| 270 | rs118142769 | - | C/T | intronic |  |  |
| 271 | rs138177673 | - | -/TCTT | intronic |  |  |
| 272 | rs138356077 | - | C/T | intronic |  |  |
| 273 | rs138756830 | - | C/T | intronic |  |  |
| 274 | rs139088397 | - | C/T | intronic |  |  |
| 275 | rs139646337 | - | C/T | intronic |  |  |
| 276 | rs139655633 | - | -/TCCGTCC | intronic |  |  |
| 277 | rs139762197 | - | C/T | intronic |  |  |
| 278 | rs140708627 | - | C/T | intronic |  |  |
| 279 | rs140790855 | - | A/G | intronic |  |  |
| 280 | rs140975443 | - | -/TGAG | intronic |  |  |
| 281 | rs141026775 | - | G/T | intronic |  |  |
| 282 | rs142037853 | - | C/T | intronic |  |  |
| 283 | rs142144999 | - | A/G | intronic |  |  |
| 284 | rs142638041 | - | A/G | intronic |  |  |
| 285 | rs142835562 | - | C/T | intronic |  |  |
| 286 | rs143706367 | - | A/G | intronic |  |  |
| 287 | rs143835533 | - | C/T | intronic |  |  |
| 288 | rs144270966 | - | A/G | intronic |  |  |
| 289 | rs145067338 | - | C/G | intronic |  |  |
| 290 | rs145169167 | - | C/T | intronic |  |  |
| 291 | rs145903125 | - | C/T | intronic |  |  |
| 292 | rs146317339 | - | -/T | intronic |  |  |
| 293 | rs146334626 | - | A/C | intronic |  |  |
| 294 | rs146840776 | - | A/G | intronic |  |  |
| 295 | rs147401611 | - | A/C | intronic |  |  |
| 296 | rs147431803 | - | C/T | intronic |  |  |
| 297 | rs148092930 | - | G/T | intronic |  |  |
| 298 | rs148146652 | - | C/T | intronic |  |  |
| 299 | rs148668179 | - | C/T | intronic |  |  |
| 300 | rs149583181 | - | C/T | intronic |  |  |
| 301 | rs149697428 | - | A/G | intronic |  |  |
| 302 | rs149750844 | - | A/G | intronic |  |  |
| 303 | rs150221300 | - | C/T | intronic |  |  |
| 304 | rs150558505 | - | A/C | intronic |  |  |
| 305 | rs180825176 | - | C/T | intronic |  |  |
| 306 | rs180919943 | - | C/G | intronic |  |  |
| 307 | rs181387336 | - | A/G | intronic |  |  |
| 308 | rs181560394 | - | A/G | intronic |  |  |
| 309 | rs181794254 | - | C/T | intronic |  |  |
| 310 | rs181983635 | - | C/G | intronic |  |  |
| 311 | rs182381541 | - | A/G | intronic |  |  |
| 312 | rs182854943 | - | A/G | intronic |  |  |
| 313 | rs182919256 | - | C/T | intronic |  |  |
| 314 | rs183593889 | - | C/T | intronic |  |  |
| 315 | rs184325284 | - | C/G | intronic |  |  |
| 316 | rs184364335 | - | C/T | intronic |  |  |
| 317 | rs184814875 | - | C/G | intronic |  |  |
| 318 | rs184844914 | - | C/T | intronic |  |  |
| 319 | rs185835539 | - | A/G | intronic |  |  |
| 320 | rs186159187 | - | C/G | intronic |  |  |
| 321 | rs186540473 | - | A/G | intronic |  |  |
| 322 | rs186603251 | - | C/T | intronic |  |  |
| 323 | rs187718007 | - | C/G | intronic |  |  |
| 324 | rs187866832 | - | C/T | intronic |  |  |
| 325 | rs189148726 | - | A/G | intronic |  |  |
| 326 | rs189164235 | - | A/G | intronic |  |  |
| 327 | rs189274237 | - | A/C | intronic |  |  |
| 328 | rs189418047 | - | C/T | intronic |  |  |
| 329 | rs189924643 | - | C/T | intronic |  |  |
| 330 | rs190019028 | - | C/T | intronic |  |  |
| 331 | rs190684432 | - | A/C | intronic |  |  |
| 332 | rs190689117 | - | A/G | intronic |  |  |
| 333 | rs190985814 | - | A/G | intronic |  |  |
| 334 | rs191781447 | - | A/G | intronic |  |  |
| 335 | rs192155835 | - | A/G | intronic |  |  |
| 336 | rs192201237 | - | A/G | intronic |  |  |
| 337 | rs192572690 | - | A/T | intronic |  |  |
| 338 | rs192646388 | - | A/T | intronic |  |  |
| 339 | rs192794013 | - | C/G | intronic |  |  |
| 340 | rs193034819 | - | A/T | intronic |  |  |
| 341 | rs199717800 | - | -/TTCT | intronic |  |  |
| 342 | rs199834505 | - | -/GGGGCGGGGCAGGAGGGA | intronic |  |  |
| 343 | rs200097824 | - | -/TCTT | intronic |  |  |
| 344 | rs200319822 | - | C/T | intronic |  |  |
| 345 | rs200687562 | - | -/GGCGGGGCAGGAGGGAGC | intronic |  |  |
| 346 | rs200728000 | - | A/G | intronic |  |  |
| 347 | rs200834188 | - | -/G | intronic |  |  |
| 348 | rs201078525 | - | G/T | intronic |  |  |
| 349 | rs201784780 | - | A/G | intronic |  |  |
| 350 | rs201880739 | - | A/C/G | intronic |  |  |
| 351 | rs202093488 | - | A/T | intronic |  |  |
| 352 | rs368551565 | - | C/T | intronic |  |  |
| 353 | rs368764025 | - | C/T | intronic |  |  |
| 354 | rs368933538 | - | C/G | intronic |  |  |
| 355 | rs368984664 | - | G/T | intronic |  |  |
| 356 | rs369420616 | - | A/G | intronic |  |  |
| 357 | rs369580088 | - | A/G | intronic |  |  |
| 358 | rs369825401 | - | A/G | intronic |  |  |
| 359 | rs370495060 | - | G/T | intronic |  |  |
| 360 | rs370508628 | - | C/T | intronic |  |  |
| 361 | rs370966747 | - | -/TGTTCCT | intronic |  |  |
| 362 | rs371088722 | - | A/G | intronic |  |  |
| 363 | rs371387738 | - | C/T | intronic |  |  |
| 364 | rs371481906 | - | C/G | intronic |  |  |
| 365 | rs371579788 | - | C/T | intronic |  |  |
| 366 | rs371632726 | - | A/G | intronic |  |  |
| 367 | rs371673816 | - | -/TGAG | intronic |  |  |
| 368 | rs371784490 | - | A/G | intronic |  |  |
| 369 | rs371895433 | - | C/T | intronic |  |  |
| 370 | rs372615034 | - | A/G | intronic |  |  |
| 371 | rs372637561 | - | C/T | intronic |  |  |
| 372 | rs372644669 | - | A/C | intronic |  |  |
| 373 | rs372891398 | - | A/G | intronic |  |  |
| 374 | rs372982994 | - | -/TTTT | intronic |  |  |
| 375 | rs373123834 | - | A/G | intronic |  |  |
| 376 | rs373458125 | - | A/G | intronic |  |  |
| 377 | rs373737457 | - | A/G | intronic |  |  |
| 378 | rs373906212 | - | A/G | intronic |  |  |
| 379 | rs373940943 | - | A/G | intronic |  |  |
| 380 | rs373961705 | - | C/G | intronic |  |  |
| 381 | rs374064351 | - | A/GGGCAGCTGG | intronic |  |  |
| 382 | rs374084935 | - | C/T | intronic |  |  |
| 383 | rs374225804 | - | C/T | intronic |  |  |
| 384 | rs374248691 | - | -/AG | intronic |  |  |
| 385 | rs374418213 | - | A/G | intronic |  |  |
| 386 | rs375039721 | - | C/T | intronic |  |  |
| 387 | rs375682593 | - | C/T | intronic |  |  |
| 388 | rs375683843 | - | C/T | intronic |  |  |
| 389 | rs375849504 | - | A/G | intronic |  |  |
| 390 | rs376307141 | - | C/T | intronic |  |  |
| 391 | rs376383649 | - | A/C/T | intronic |  |  |
| 392 | rs376386421 | - | C/T | intronic |  |  |
| 393 | rs376827085 | - | A/C | intronic |  |  |
| 394 | rs376931241 | - | C/T | intronic |  |  |
| 395 | rs377018212 | - | C/G | intronic |  |  |
| 396 | rs377188753 | - | C/T | intronic |  |  |
| 397 | rs377242552 | - | G/T | intronic |  |  |
| 398 | rs377402141 | - | A/G | intronic |  |  |
| 399 | rs377508111 | - | G/T | intronic |  |  |
| 400 | rs377738276 | - | -/T | intronic |  |  |
| 401 | rs377746594 | - | -/TGT | intronic |  |  |
| 402 | rs397731916 | - | -/G | intronic |  |  |
| 403 | rs527246607 | - | C/T | intronic |  |  |
| 404 | rs527541915 | - | A/G | intronic |  |  |
| 405 | rs527841736 | - | C/T | intronic |  |  |
| 406 | rs528297617 | - | C/G | intronic |  |  |
| 407 | rs528565386 | - | A/G | intronic |  |  |
| 408 | rs529386791 | - | A/G | intronic |  |  |
| 409 | rs529405437 | - | A/G | intronic |  |  |
| 410 | rs529427465 | - | G/T | intronic |  |  |
| 411 | rs529813568 | - | C/T | intronic |  |  |
| 412 | rs530262987 | - | A/G | intronic |  |  |
| 413 | rs530769794 | - | C/T | intronic |  |  |
| 414 | rs531236765 | - | C/T | intronic |  |  |
| 415 | rs531303016 | - | G/T | intronic |  |  |
| 416 | rs531829108 | - | A/G | intronic |  |  |
| 417 | rs532058134 | - | G/T | intronic |  |  |
| 418 | rs532205584 | - | C/T | intronic |  |  |
| 419 | rs532718233 | - | G/T | intronic |  |  |
| 420 | rs532843256 | - | A/G | intronic |  |  |
| 421 | rs533394871 | - | G/T | intronic |  |  |
| 422 | rs533734130 | - | C/T | intronic |  |  |
| 423 | rs533865683 | - | A/G | intronic |  |  |
| 424 | rs533973625 | - | C/T | intronic |  |  |
| 425 | rs534033010 | - | A/G | intronic |  |  |
| 426 | rs534081374 | - | A/G | intronic |  |  |
| 427 | rs534354828 | - | C/T | intronic |  |  |
| 428 | rs534716473 | - | A/G | intronic |  |  |
| 429 | rs535216959 | - | C/T | intronic |  |  |
| 430 | rs535271865 | - | A/G | intronic |  |  |
| 431 | rs535751335 | - | A/G | intronic |  |  |
| 432 | rs535821311 | - | C/T | intronic |  |  |
| 433 | rs535846356 | - | A/G | intronic |  |  |
| 434 | rs536281496 | - | C/G | intronic |  |  |
| 435 | rs536354933 | - | C/T | intronic |  |  |
| 436 | rs536418682 | - | C/G | intronic |  |  |
| 437 | rs536820228 | - | A/C | intronic |  |  |
| 438 | rs537162473 | - | C/T | intronic |  |  |
| 439 | rs537312361 | - | -/GA | intronic |  |  |
| 440 | rs537347112 | - | A/G | intronic |  |  |
| 441 | rs537679194 | - | C/T | intronic |  |  |
| 442 | rs537752213 | - | G/T | intronic |  |  |
| 443 | rs538208762 | - | A/G | intronic |  |  |
| 444 | rs538227700 | - | C/T | intronic |  |  |
| 445 | rs538351224 | - | A/G | intronic |  |  |
| 446 | rs538959548 | - | G/T | intronic |  |  |
| 447 | rs539196352 | - | A/C | intronic |  |  |
| 448 | rs539534374 | - | C/T | intronic |  |  |
| 449 | rs540010509 | - | C/T | intronic |  |  |
| 450 | rs540024299 | - | -/TC | intronic |  |  |
| 451 | rs540091191 | - | A/T | intronic |  |  |
| 452 | rs540152148 | - | A/G | intronic |  |  |
| 453 | rs540171463 | - | C/T | intronic |  |  |
| 454 | rs540212669 | - | G/T | intronic |  |  |
| 455 | rs540709795 | - | C/G | intronic |  |  |
| 456 | rs541492091 | - | C/T | intronic |  |  |
| 457 | rs541555353 | - | C/T | intronic |  |  |
| 458 | rs541618537 | - | A/G | intronic |  |  |
| 459 | rs541688578 | - | C/T | intronic |  |  |
| 460 | rs542021689 | - | A/G | intronic |  |  |
| 461 | rs542067947 | - | C/T | intronic |  |  |
| 462 | rs542084995 | - | C/T | intronic |  |  |
| 463 | rs543047037 | - | G/T | intronic |  |  |
| 464 | rs543305650 | - | A/G | intronic |  |  |
| 465 | rs543577717 | - | A/G | intronic |  |  |
| 466 | rs543706810 | - | A/G | intronic |  |  |
| 467 | rs544128172 | - | C/T | intronic |  |  |
| 468 | rs544185829 | - | C/T | intronic |  |  |
| 469 | rs544376621 | - | C/G | intronic |  |  |
| 470 | rs544524626 | - | C/G | intronic |  |  |
| 471 | rs545026366 | - | C/T | intronic |  |  |
| 472 | rs545055615 | - | A/T | intronic |  |  |
| 473 | rs545589186 | - | A/G | intronic |  |  |
| 474 | rs545862196 | - | C/T | intronic |  |  |
| 475 | rs546126072 | - | C/T | intronic |  |  |
| 476 | rs546133056 | - | C/G | intronic |  |  |
| 477 | rs546372127 | - | C/T | intronic |  |  |
| 478 | rs546380166 | - | C/G | intronic |  |  |
| 479 | rs546485652 | - | C/G | intronic |  |  |
| 480 | rs547741987 | - | C/T | intronic |  |  |
| 481 | rs547804156 | - | C/G | intronic |  |  |
| 482 | rs547929933 | - | A/G | intronic |  |  |
| 483 | rs547994320 | - | A/G | intronic |  |  |
| 484 | rs548186143 | - | -/GGGC | intronic |  |  |
| 485 | rs548201426 | - | A/G | intronic |  |  |
| 486 | rs548218742 | - | A/G | intronic |  |  |
| 487 | rs548365996 | - | C/T | intronic |  |  |
| 488 | rs548429736 | - | A/G | intronic |  |  |
| 489 | rs548754913 | - | C/T | intronic |  |  |
| 490 | rs548957298 | - | C/T | intronic |  |  |
| 491 | rs549053153 | - | G/T | intronic |  |  |
| 492 | rs549274445 | - | C/T | intronic |  |  |
| 493 | rs549523738 | - | C/T | intronic |  |  |
| 494 | rs550128384 | - | C/T | intronic |  |  |
| 495 | rs550303992 | - | C/T | intronic |  |  |
| 496 | rs550408514 | - | C/T | intronic |  |  |
| 497 | rs550489896 | - | G/T | intronic |  |  |
| 498 | rs550513638 | - | C/G | intronic |  |  |
| 499 | rs550611194 | - | C/T | intronic |  |  |
| 500 | rs550650273 | - | G/T | intronic |  |  |
| 501 | rs551215389 | - | A/G | intronic |  |  |
| 502 | rs551330816 | - | -/GCTGG | intronic |  |  |
| 503 | rs551367550 | - | C/T | intronic |  |  |
| 504 | rs551678342 | - | G/T | intronic |  |  |
| 505 | rs551724408 | - | A/G | intronic |  |  |
| 506 | rs552536846 | - | C/T | intronic |  |  |
| 507 | rs552608282 | - | C/G | intronic |  |  |
| 508 | rs552628902 | - | C/T | intronic |  |  |
| 509 | rs552671583 | - | A/G | intronic |  |  |
| 510 | rs552741467 | - | C/T | intronic |  |  |
| 511 | rs553285312 | - | C/T | intronic |  |  |
| 512 | rs553556885 | - | -/A | intronic |  |  |
| 513 | rs553599805 | - | C/G | intronic |  |  |
| 514 | rs554005948 | - | A/C | intronic |  |  |
| 515 | rs555137754 | - | A/G | intronic |  |  |
| 516 | rs555200954 | - | A/C | intronic |  |  |
| 517 | rs555505493 | - | C/T | intronic |  |  |
| 518 | rs555527837 | - | C/G | intronic |  |  |
| 519 | rs555751218 | - | -/G | intronic |  |  |
| 520 | rs556000404 | - | A/G | intronic |  |  |
| 521 | rs556056869 | - | C/G | intronic |  |  |
| 522 | rs556210991 | - | C/G | intronic |  |  |
| 523 | rs556465131 | - | C/G | intronic |  |  |
| 524 | rs556511165 | - | C/T | intronic |  |  |
| 525 | rs556741053 | - | C/T | intronic |  |  |
| 526 | rs557002398 | - | A/G | intronic |  |  |
| 527 | rs557263615 | - | C/G | intronic |  |  |
| 528 | rs557391136 | - | A/T | intronic |  |  |
| 529 | rs557456493 | - | A/G | intronic |  |  |
| 530 | rs557539718 | - | C/T | intronic |  |  |
| 531 | rs557930449 | - | G/T | intronic |  |  |
| 532 | rs557946856 | - | A/C/T | intronic |  |  |
| 533 | rs558527684 | - | C/T | intronic |  |  |
| 534 | rs558584194 | - | C/T | intronic |  |  |
| 535 | rs558786166 | - | A/G | intronic |  |  |
| 536 | rs559860152 | - | C/T | intronic |  |  |
| 537 | rs559860211 | - | C/T | intronic |  |  |
| 538 | rs559922325 | - | A/G | intronic |  |  |
| 539 | rs560201706 | - | A/G | intronic |  |  |
| 540 | rs560447242 | - | C/T | intronic |  |  |
| 541 | rs560518129 | - | C/T | intronic |  |  |
| 542 | rs560888620 | - | C/T | intronic |  |  |
| 543 | rs561282203 | - | -/ATCTGGGAG | intronic |  |  |
| 544 | rs561337851 | - | A/G | intronic |  |  |
| 545 | rs561728652 | - | A/G | intronic |  |  |
| 546 | rs562205702 | - | A/T | intronic |  |  |
| 547 | rs562311458 | - | C/T | intronic |  |  |
| 548 | rs562482045 | - | A/C | intronic |  |  |
| 549 | rs562543385 | - | C/T | intronic |  |  |
| 550 | rs562763758 | - | C/T | intronic |  |  |
| 551 | rs562765259 | - | C/T | intronic |  |  |
| 552 | rs562818886 | - | C/T | intronic |  |  |
| 553 | rs562881933 | - | C/T | intronic |  |  |
| 554 | rs563535145 | - | A/G | intronic |  |  |
| 555 | rs563701714 | - | C/T | intronic |  |  |
| 556 | rs564292620 | - | C/G | intronic |  |  |
| 557 | rs564404579 | - | C/T | intronic |  |  |
| 558 | rs564810687 | - | A/G | intronic |  |  |
| 559 | rs565481174 | - | A/C | intronic |  |  |
| 560 | rs565966125 | - | A/G | intronic |  |  |
| 561 | rs566091388 | - | A/G | intronic |  |  |
| 562 | rs566480932 | - | A/C | intronic |  |  |
| 563 | rs566490686 | - | -/TT | intronic |  |  |
| 564 | rs566580110 | - | C/T | intronic |  |  |
| 565 | rs566608218 | - | C/T | intronic |  |  |
| 566 | rs566672863 | - | C/G | intronic |  |  |
| 567 | rs566735280 | - | C/T | intronic |  |  |
| 568 | rs567004162 | - | C/G | intronic |  |  |
| 569 | rs567132675 | - | A/G | intronic |  |  |
| 570 | rs567460961 | - | C/T | intronic |  |  |
| 571 | rs568004127 | - | A/G | intronic |  |  |
| 572 | rs568008390 | - | A/T | intronic |  |  |
| 573 | rs568173544 | - | C/T | intronic |  |  |
| 574 | rs568347907 | - | C/T | intronic |  |  |
| 575 | rs568501993 | - | C/T | intronic |  |  |
| 576 | rs568777729 | - | A/G | intronic |  |  |
| 577 | rs568840878 | - | A/C | intronic |  |  |
| 578 | rs568850846 | - | A/C | intronic |  |  |
| 579 | rs568868318 | - | A/G | intronic |  |  |
| 580 | rs568882257 | - | C/T | intronic |  |  |
| 581 | rs568983750 | - | A/G | intronic |  |  |
| 582 | rs569366596 | - | C/T | intronic |  |  |
| 583 | rs569513096 | - | C/T | intronic |  |  |
| 584 | rs569825816 | - | A/C | intronic |  |  |
| 585 | rs569851168 | - | A/G | intronic |  |  |
| 586 | rs569945979 | - | A/C | intronic |  |  |
| 587 | rs570241682 | - | A/C | intronic |  |  |
| 588 | rs570842703 | - | A/G | intronic |  |  |
| 589 | rs571119344 | - | A/G | intronic |  |  |
| 590 | rs571211834 | - | C/T | intronic |  |  |
| 591 | rs571888611 | - | A/T | intronic |  |  |
| 592 | rs572223021 | - | C/T | intronic |  |  |
| 593 | rs572350234 | - | C/T | intronic |  |  |
| 594 | rs572664115 | - | A/G | intronic |  |  |
| 595 | rs572797970 | - | A/G | intronic |  |  |
| 596 | rs572860883 | - | A/G | intronic |  |  |
| 597 | rs572987536 | - | A/G | intronic |  |  |
| 598 | rs573080130 | - | A/G | intronic |  |  |
| 599 | rs573263800 | - | A/G | intronic |  |  |
| 600 | rs573321353 | - | A/C | intronic |  |  |
| 601 | rs573697853 | - | A/G | intronic |  |  |
| 602 | rs573722475 | - | C/T | intronic |  |  |
| 603 | rs574052443 | - | C/T | intronic |  |  |
| 604 | rs574237762 | - | A/C | intronic |  |  |
| 605 | rs574309140 | - | C/G | intronic |  |  |
| 606 | rs574646673 | - | C/T | intronic |  |  |
| 607 | rs575552554 | - | A/G | intronic |  |  |
| 608 | rs575579736 | - | C/T | intronic |  |  |
| 609 | rs575739954 | - | C/T | intronic |  |  |
| 610 | rs576014966 | - | A/G | intronic |  |  |
| 611 | rs576080160 | - | C/T | intronic |  |  |
| 612 | rs576322275 | - | A/G | intronic |  |  |
| 613 | rs576605476 | - | A/G | intronic |  |  |
| 614 | rs576655249 | - | C/T | intronic |  |  |
| 615 | rs576989499 | - | C/T | intronic |  |  |
| 616 | rs577169601 | - | C/G | intronic |  |  |
| 617 | rs577234383 | - | A/G | intronic |  |  |
| 618 | rs577847887 | - | C/G | intronic |  |  |
| 619 | rs578025139 | - | A/G | intronic |  |  |
| 620 | rs578087220 | - | A/G | intronic |  |  |
| 621 | rs745443260 | - | A/G | intronic |  |  |
| 622 | rs745551855 | - | C/T | intronic |  |  |
| 623 | rs745762364 | - | C/T | intronic |  |  |
| 624 | rs745778320 | - | A/G | intronic |  |  |
| 625 | rs746005390 | - | A/C | intronic |  |  |
| 626 | rs746384947 | - | A/G | intronic |  |  |
| 627 | rs746605782 | - | C/T | intronic |  |  |
| 628 | rs746757983 | - | A/G | intronic |  |  |
| 629 | rs746884894 | - | A/G | intronic |  |  |
| 630 | rs747956607 | - | A/G | intronic |  |  |
| 631 | rs748303765 | - | A/G | intronic |  |  |
| 632 | rs748404984 | - | -/A | intronic |  |  |
| 633 | rs748592590 | - | C/T | intronic |  |  |
| 634 | rs748725018 | - | A/G | intronic |  |  |
| 635 | rs748778969 | - | C/G | intronic |  |  |
| 636 | rs749497035 | - | C/T | intronic |  |  |
| 637 | rs749680649 | - | C/T | intronic |  |  |
| 638 | rs749824230 | - | C/T | intronic |  |  |
| 639 | rs750349204 | - | C/T | intronic |  |  |
| 640 | rs750947514 | - | C/G | intronic |  |  |
| 641 | rs750950632 | - | A/G | intronic |  |  |
| 642 | rs750994845 | - | C/G/T | intronic |  |  |
| 643 | rs751164399 | - | G/T | intronic |  |  |
| 644 | rs751563551 | - | A/G | intronic |  |  |
| 645 | rs751718568 | - | A/G | intronic |  |  |
| 646 | rs751813711 | - | C/T | intronic |  |  |
| 647 | rs751910716 | - | A/G | intronic |  |  |
| 648 | rs751972738 | - | C/G | intronic |  |  |
| 649 | rs752169927 | - | -/TTCT | intronic |  |  |
| 650 | rs752334560 | - | A/G | intronic |  |  |
| 651 | rs752385147 | - | C/T | intronic |  |  |
| 652 | rs752527443 | - | A/G | intronic |  |  |
| 653 | rs752664052 | - | A/G | intronic |  |  |
| 654 | rs753149657 | - | A/T | intronic |  |  |
| 655 | rs753235904 | - | C/T | intronic |  |  |
| 656 | rs753398897 | - | A/C | intronic |  |  |
| 657 | rs753441848 | - | A/G | intronic |  |  |
| 658 | rs753659908 | - | C/G | intronic |  |  |
| 659 | rs753849621 | - | C/T | intronic |  |  |
| 660 | rs754009783 | - | A/T | intronic |  |  |
| 661 | rs754041716 | - | A/G | intronic |  |  |
| 662 | rs754728465 | - | C/T | intronic |  |  |
| 663 | rs754817647 | - | A/G | intronic |  |  |
| 664 | rs754840815 | - | A/G | intronic |  |  |
| 665 | rs755760336 | - | C/T | intronic |  |  |
| 666 | rs755898393 | - | C/T | intronic |  |  |
| 667 | rs756210195 | - | C/T | intronic |  |  |
| 668 | rs756386013 | - | A/G | intronic |  |  |
| 669 | rs756594493 | - | C/G | intronic |  |  |
| 670 | rs756757951 | - | A/G | intronic |  |  |
| 671 | rs757089049 | - | G/T | intronic |  |  |
| 672 | rs757422738 | - | A/G | intronic |  |  |
| 673 | rs757566726 | - | A/G | intronic |  |  |
| 674 | rs757999343 | - | G/T | intronic |  |  |
| 675 | rs758128755 | - | C/T | intronic |  |  |
| 676 | rs758602421 | - | C/T | intronic |  |  |
| 677 | rs758754802 | - | A/G | intronic |  |  |
| 678 | rs758845609 | - | C/T | intronic |  |  |
| 679 | rs758948952 | - | A/T | intronic |  |  |
| 680 | rs759332329 | - | C/T | intronic |  |  |
| 681 | rs759688508 | - | -/A | intronic |  |  |
| 682 | rs759952816 | - | A/T | intronic |  |  |
| 683 | rs760142706 | - | C/T | intronic |  |  |
| 684 | rs760158292 | - | C/T | intronic |  |  |
| 685 | rs760322912 | - | C/T | intronic |  |  |
| 686 | rs760475679 | - | A/T | intronic |  |  |
| 687 | rs760878391 | - | A/G | intronic |  |  |
| 688 | rs760894949 | - | C/T | intronic |  |  |
| 689 | rs761157213 | - | A/C | intronic |  |  |
| 690 | rs761574724 | - | A/G | intronic |  |  |
| 691 | rs761635136 | - | C/G | intronic |  |  |
| 692 | rs761915112 | - | C/T | intronic |  |  |
| 693 | rs762233285 | - | C/T | intronic |  |  |
| 694 | rs762240782 | - | C/T | intronic |  |  |
| 695 | rs762322978 | - | A/G | intronic |  |  |
| 696 | rs762365896 | - | C/T | intronic |  |  |
| 697 | rs762491836 | - | A/G | intronic |  |  |
| 698 | rs762588689 | - | A/G | intronic |  |  |
| 699 | rs762599779 | - | A/G | intronic |  |  |
| 700 | rs763324217 | - | C/T | intronic |  |  |
| 701 | rs763364246 | - | A/G | intronic |  |  |
| 702 | rs763388553 | - | -/TGAG | intronic |  |  |
| 703 | rs763567442 | - | C/T | intronic |  |  |
| 704 | rs763598359 | - | C/T | intronic |  |  |
| 705 | rs763799662 | - | C/T | intronic |  |  |
| 706 | rs763969577 | - | C/T | intronic |  |  |
| 707 | rs764229144 | - | A/C | intronic |  |  |
| 708 | rs764486066 | - | A/G | intronic |  |  |
| 709 | rs764762982 | - | A/G | intronic |  |  |
| 710 | rs764839101 | - | A/G | intronic |  |  |
| 711 | rs764912038 | - | A/G | intronic |  |  |
| 712 | rs765034470 | - | C/T | intronic |  |  |
| 713 | rs765510441 | - | A/G | intronic |  |  |
| 714 | rs765728030 | - | A/G | intronic |  |  |
| 715 | rs765853866 | - | C/T | intronic |  |  |
| 716 | rs765921282 | - | A/G | intronic |  |  |
| 717 | rs766011273 | - | A/G | intronic |  |  |
| 718 | rs766313115 | - | A/C | intronic |  |  |
| 719 | rs766666503 | - | A/G | intronic |  |  |
| 720 | rs766754780 | - | C/T | intronic |  |  |
| 721 | rs766940905 | - | C/T | intronic |  |  |
| 722 | rs767132242 | - | A/G | intronic |  |  |
| 723 | rs767322875 | - | A/G | intronic |  |  |
| 724 | rs767866004 | - | A/G | intronic |  |  |
| 725 | rs768067983 | - | -/TT | intronic |  |  |
| 726 | rs768202597 | - | A/G | intronic |  |  |
| 727 | rs768239360 | - | -/CT | intronic |  |  |
| 728 | rs768464082 | - | A/G | intronic |  |  |
| 729 | rs768705662 | - | -/T | intronic |  |  |
| 730 | rs768771280 | - | C/T | intronic |  |  |
| 731 | rs768796658 | - | C/G | intronic |  |  |
| 732 | rs768899649 | - | C/T | intronic |  |  |
| 733 | rs769034928 | - | A/C | intronic |  |  |
| 734 | rs769743065 | - | G/T | intronic |  |  |
| 735 | rs769914892 | - | A/C | intronic |  |  |
| 736 | rs770007222 | - | -/TCCCTGA | intronic |  |  |
| 737 | rs770273145 | - | C/T | intronic |  |  |
| 738 | rs770364413 | - | -/TTTA | intronic |  |  |
| 739 | rs770467587 | - | C/T | intronic |  |  |
| 740 | rs770801880 | - | C/G | intronic |  |  |
| 741 | rs770870580 | - | -/TGCTGAATGG | intronic |  |  |
| 742 | rs770880317 | - | C/T | intronic |  |  |
| 743 | rs771074121 | - | C/G | intronic |  |  |
| 744 | rs771190871 | - | A/G | intronic |  |  |
| 745 | rs771326115 | - | A/G | intronic |  |  |
| 746 | rs771487582 | - | -/G | intronic |  |  |
| 747 | rs771504581 | - | C/T | intronic |  |  |
| 748 | rs771735915 | - | C/T | intronic |  |  |
| 749 | rs771942072 | - | C/G | intronic |  |  |
| 750 | rs772557687 | - | -/C | intronic |  |  |
| 751 | rs772670189 | - | C/T | intronic |  |  |
| 752 | rs772848455 | - | A/G | intronic |  |  |
| 753 | rs772901541 | - | C/T | intronic |  |  |
| 754 | rs773289840 | - | G/T | intronic |  |  |
| 755 | rs773457838 | - | -/CA | intronic |  |  |
| 756 | rs773580725 | - | C/T | intronic |  |  |
| 757 | rs773783515 | - | C/G | intronic |  |  |
| 758 | rs774238168 | - | C/T | intronic |  |  |
| 759 | rs774247005 | - | A/G | intronic |  |  |
| 760 | rs774904653 | - | C/T | intronic |  |  |
| 761 | rs775008206 | - | C/T | intronic |  |  |
| 762 | rs775263818 | - | A/G | intronic |  |  |
| 763 | rs775674163 | - | C/T | intronic |  |  |
| 764 | rs775692483 | - | C/T | intronic |  |  |
| 765 | rs775741310 | - | C/T | intronic |  |  |
| 766 | rs775950233 | - | C/T | intronic |  |  |
| 767 | rs775971082 | - | A/G | intronic |  |  |
| 768 | rs776074539 | - | A/T | intronic |  |  |
| 769 | rs776249439 | - | C/T | intronic |  |  |
| 770 | rs776953146 | - | G/T | intronic |  |  |
| 771 | rs777083445 | - | C/T | intronic |  |  |
| 772 | rs777431441 | - | C/G | intronic |  |  |
| 773 | rs777656218 | - | A/C | intronic |  |  |
| 774 | rs777892439 | - | C/T | intronic |  |  |
| 775 | rs777916643 | - | A/G | intronic |  |  |
| 776 | rs778276873 | - | G/T | intronic |  |  |
| 777 | rs778899433 | - | -/T | intronic |  |  |
| 778 | rs778946143 | - | A/G | intronic |  |  |
| 779 | rs779089811 | - | G/T | intronic |  |  |
| 780 | rs779133076 | - | A/G | intronic |  |  |
| 781 | rs779207261 | - | C/T | intronic |  |  |
| 782 | rs779332955 | - | C/T | intronic |  |  |
| 783 | rs779405133 | - | C/G | intronic |  |  |
| 784 | rs779567122 | - | A/T | intronic |  |  |
| 785 | rs779826716 | - | A/G | intronic |  |  |
| 786 | rs779862376 | - | A/G | intronic |  |  |
| 787 | rs780057400 | - | C/G | intronic |  |  |
| 788 | rs780351612 | - | -/TTTA | intronic |  |  |
| 789 | rs780505414 | - | C/T | intronic |  |  |
| 790 | rs780876845 | - | C/G | intronic |  |  |
| 791 | rs780898209 | - | A/G | intronic |  |  |
| 792 | rs781077773 | - | C/T | intronic |  |  |
| 793 | rs781166269 | - | A/G | intronic |  |  |
| 794 | rs781286079 | - | G/T | intronic |  |  |
| 795 | rs781617447 | - | A/G | intronic |  |  |
| 796 | rs796303731 | - | C/T | intronic |  |  |
| 797 | rs796363454 | - | AC/TT | intronic |  |  |
| 798 | rs796394821 | - | A/C | intronic |  |  |
| 799 | rs796989477 | - | -/T | intronic |  |  |
| 800 | rs866412722 | - | A/G | intronic |  |  |
| 801 | rs866698065 | - | C/T | intronic |  |  |
| 802 | rs866747433 | - | C/T | intronic |  |  |
| 803 | rs867016247 | - | C/T | intronic |  |  |
| 804 | rs867162745 | - | C/G | intronic |  |  |
| 805 | rs867245233 | - | C/T | intronic |  |  |
| 806 | rs867336465 | - | C/T | intronic |  |  |
| 807 | rs867496184 | - | G/T | intronic |  |  |
| 808 | rs867574750 | - | C/T | intronic |  |  |
| 809 | rs867588665 | - | A/G | intronic |  |  |
| 810 | rs867588972 | - | A/C | intronic |  |  |
| 811 | rs867721175 | - | G/T | intronic |  |  |
| 812 | rs868060402 | - | G/T | intronic |  |  |
| 813 | rs868175087 | - | C/G | intronic |  |  |
| 814 | rs868196009 | - | C/T | intronic |  |  |
| 815 | rs868221033 | - | C/T | intronic |  |  |
| 816 | rs868483960 | - | C/T | intronic |  |  |
| 817 | rs868681540 | - | A/G | intronic |  |  |
| 818 | rs868823041 | - | C/G | intronic |  |  |
