# Supplementary material for: Effects of genetic variants in the TSPO gene on protein structure and stability
Source: PLoS One. 2018 Apr 11;13(4):e0195627. doi: 10.1371/journal.pone.0195627 (PMC5895031; doi:10.1371/journal.pone.0195627)
Supplement: S2 Table — (DOCX) [file pone.0195627.s005.docx]

**Supplementary Table 2. The summary of ConSurf analysis and the putative deleterious TSPO nsSNPs**

| a.a. residue | score | conservation score |
| --- | --- | --- |
|  |  |  |
| W5R | 0.572 | 3 |
| G10S | 0.967 | 2 |
| L13P | 0.737 | 2 |
| G22A | -0.118 | 5 |
| G30D | 0.257 | 4  **TMD2** |
| R32C | 1.298 | 1 |
| **P44L** | **-1.134** | **9** |
| **G63S** | **-0.54** | **7** |
| **G63D** | **-0.54** | **7** |
| **Y65F** | **-1.097** | **9** |
| G83R | 0.468 | 3 |
| **L84V** | **-0.324** | **6** |
| **P97L** | **-0.285** | **6** |
| A120G | 0.477 | 3  **TMD5** |
| P131L | 1.057 | 1 |
| **A133T** | **-1.203** | **9** |
| **P139T** | **-1.22** | **9** |
| **P139S** | **-1.22** | **9** |
| **A147T** | **-1.128** | **9** |
| **L150F** | **-1.066** | **9** |
| R156W | 0.364 | 4 |
