# Supplementary material for: Effects of genetic variants in the TSPO gene on protein structure and stability
Source: PLoS One. 2018 Apr 11;13(4):e0195627. doi: 10.1371/journal.pone.0195627 (PMC5895031; doi:10.1371/journal.pone.0195627)
Supplement: S3 Table — (DOCX) [file pone.0195627.s006.docx]

**Supplementary Table 3.** **Comparison of** **codon relative adaptiveness values for wt and sSNPs**

| sSNP position | **wt*** | **wt codon** | **sSNP*** |
| --- | --- | --- | --- |
| P7 | 100 | CCC | 91 |
| A14 | 28 | GCG | 57 |
| P15 | 100 | CCC | 91 |
| F20 | 100 | TTC | 85 |
| S23 | 100 | TCC | 21 |
| V26 | 52 | GTC | 39 |
| G28 | 100 | GGC | 47 |
| G30 | 47 | GGT | 100 |
| L31 | 50 | CTC | 33 |
| Y34 | 100 | TAC | 79 |
| A35 | 100 | GCC | 68 |
| S41 | 21 | TCG | 63 |
| P44 | 34 | CCG | 88 |
| T55 | 31 | ACG | 78 |
| Y62 | 100 | TAC | 69 |
| S64 | 92 | TCC | 79 |
| K77 | 100 | AAG | 75 |
| Y85 | 100 | TAC | 79 |
| Q88 | 100 | CAG | 37 |
| P96 | 100 | CCC | 88 |
| L109 | 33 | TTG | 20 |
| **L113** | **100** | **CTG** | **18** |
| **L114** | **100** | **CTG** | **18** |
| A118 | 28 | GCG | 57 |
| A119 | 28 | GCG | 57 |
| A120 | 57 | GCA | 100 |
| T123 | 100 | ACC | 69 |
| A125 | 100 | GCC | 28 |
| Y127 | 100 | TAC | 69 |
| V129 | 100 | GTG | 26 |
| A133 | 100 | GCC | 68 |
| R135 | 86 | CGC | 95 |
| L137 | 50 | CTC | 100 |
| **L141** | **100** | **CTG** | **18** |
| L144 | 100 | CTG | 33 |
| A145 | 100 | GCC | 68 |
| F146 | 100 | TTC | 85 |
| T147 | 31 | GCG | 78 |
| T148 | 100 | ACC | 69 |
| C153 | 100 | TGC | 85 |

* Codon usage is represented using the relative adaptiveness
