# Supplementary material for: Effects of genetic variants in the TSPO gene on protein structure and stability
Source: PLoS One. 2018 Apr 11;13(4):e0195627. doi: 10.1371/journal.pone.0195627 (PMC5895031; doi:10.1371/journal.pone.0195627)
Supplement: S4 Table — (DOCX) [file pone.0195627.s007.docx]

**Supplementary Table 4.** Primer sets used for sequencing

| Primer name | Forward primer (5´-3´) | Reverse primer (5´-3´) |
| --- | --- | --- |
| TSPO-ex1 | GAGGTGGCTTTGAGGAGTGA | GCAGGGTTGTCCAAGTTTTC |
| TSPO-ex2 | CTGGAAATGCGTTCACTCAG | GCCTGGAGAAGACCCTCTGT |
| TSPO-ex3 | GAAGCACTGCCAATGTGCTA | GCTTCGTGTGGGTTTTCCTA |
| TSPO-ex4 | AGTTGGGCAGTGGGACAG | GCAGATCCTGCAGAGACGA |
